# Modeling discourse structure with 2D similarity-based random walks for improved understanding of online conversations

**DOI:** 10.1038/s41598-026-43577-7

**Published:** 2026-03-12

**Authors:** Zaid Almahmoud, Vibhor Agarwal, Rana Mahmoud, Nishanth Sastry

**Affiliations:** 1 Artificial Intelligence and Media Lab (AIM-LAB), Northwestern University in Qatar, Doha, Qatar; 2 Department of Computer Science, University of Surrey, Guildford, UK; 3 Engineering Technology and Science, Higher Colleges of Technology, Abu Dhabi, United Arab Emirates

**Keywords:** Mathematics and computing, Computer science

## Abstract

The proliferation of social media has made automated classification of online discourse, such as hate speech detection and polarity prediction, an essential task for maintaining digital safety and constructive discussions. However, online conversations are complex, context-dependent, and often structured in branching discourse trees, making such tasks especially challenging. Existing classification models typically rely on limited context sampling strategies that overlook the broader structure of discussions and the semantic relevance to the target utterance. In this paper, we introduce the 2D Similarity-based Random Walk, a novel context sampling method that explores multiple paths within discourse graphs to capture more comprehensive contextual information. Through empirical analysis, we show that the proposed 2D-walk covers a wider range of branches in the discourse compared to the traditional single-path (1D) baseline, resulting in samples that are structurally richer and contain a larger number of utterances. We evaluate our method on two benchmark datasets: Guest, for detecting misogynistic hate speech, and Kialo, for predicting the polarity of argumentative replies. Extensive experiments using GPT-4, a Multi-Head Attention model, and BERT confirm that 2D-walk consistently improves classification performance across both datasets compared to several baselines, including the 1D-walk, the no-context (0D) setting, and the random sampling strategy. Our findings underscore the importance of discourse-aware sampling and suggest that leveraging both structural and semantic relationships in conversation graphs is key to advancing robust language understanding in social media contexts.

## Introduction

Online discussions shape public opinion, influence decision-making, and drive societal change^1^. However, with the exponential rise of social media engagement, identifying and understanding the nature of user-generated content–especially hate speech and sentiment polarity has become a pressing challenge^2–4^. The need for accurate, context-aware classification methods is more critical than ever, as traditional keyword-based or shallow machine learning approaches struggle to capture the nuanced relationships between utterances in dynamic conversations^5–8^. Today, NLP has progressed to the point where large language models (LLMs) can transform how we approach language understanding. These models offer unprecedented capacity for contextual reasoning, but their effectiveness depends heavily on receiving the right context in the right form. Without it, even the most powerful models can misinterpret intent, nuance, or relational meaning^9^.

Hate speech detection, particularly in the context of misogyny and gender-based hostility, has emerged as a high-stakes task for ensuring safe and inclusive digital spaces^10–12^. It involves identifying content that expresses hatred or promotes violence against individuals or groups based on characteristics such as gender, race, or religion. Despite increasing research attention, this problem remains difficult due to the subtle and context-dependent nature of hateful language, including sarcasm, coded language, and implicit bias^13^. Datasets like Guest^11^ have been curated to address this challenge by providing annotated social media discussions where hate speech is labeled in context, enabling researchers to explore not only what is said but how it relates to surrounding dialogue. Most recent approaches rely on transformer-based models^2,4^, but performance can be improved by advancing context sampling strategies.

In contrast to hate speech detection, polarity prediction focuses on assessing the stance of an utterance in relation to another. Specifically, it classifies whether a comment supports or attacks the utterance it is replying to. Kialo^14^, a platform for structured online debate, offers rich data for studying this task^15^. Each contribution in Kialo is linked to others through a tree-like argument structure, and each node expresses either a supportive or opposing stance relative to its parent. This makes polarity prediction a task of understanding semantic orientation in argumentative discourse, which is inherently relational and context-sensitive. Classifying such utterances accurately requires models to interpret how statements relate not just to their immediate context but to the broader argumentative flow of the discussion. Unlike typical sentiment analysis on product reviews or tweets, this task requires deeper contextual modeling of structured discourse.

Overall, both hate speech detection and polarity prediction highlight the critical need for models that can reason across discourse structure and semantic relationships. A central problem is that the context necessary to interpret online conversations is embedded in the structure of the discussion tree and this context is not always limited to direct parents or ancestors. In many cases, sibling or cousin comments provide essential cues for understanding the meaning or intent of a reply. For instance, detecting hate speech in a seemingly neutral comment may require awareness of sarcastic exchanges between peer-level comments. A comment like “She’s one of the good ones,” may not appear hateful on its own, but when viewed alongside sibling comments expressing racist stereotypes, its implicit bias becomes clearer. Similarly, in polarity prediction tasks such as those based on Kialo debates, sibling comments often offer semantically closer and more relevant context than distant parent or ancestor nodes. Since all sibling contributions respond to the same parent argument, they typically engage with similar aspects of the discussion, using comparable terminology or framing. For example, consider a parent argument stating “Government policies must prioritize public safety.” Several levels down, a reply narrows the discussion with, “Mass surveillance helps prevent terrorism,” and a sibling comment states, “Surveillance tools enable authorities to monitor suspicious networks effectively.” When classifying the polarity of the last comment, the broad ancestor argument about public safety offers little semantic guidance. In contrast, the sibling’s focus on surveillance aligns more closely with the target comment’s framing and critical tone, making it a far stronger and more relevant contextual signal. This highlights how semantically related sibling comments can in some cases provide more precise cues for polarity classification than more abstract or distant ancestors. Effective context modeling in online discourse should therefore consider both hierarchical (parent/ancestor) and lateral (sibling/cousin) relationships to capture the most relevant and informative context for classification tasks.

However, most existing approaches fail to capture the full discourse structure. Many either ignore context entirely^16^ or include only immediate context^17^, while others follow a single path at a time through the conversation tree to capture context^2,15^, which inherently restricts the extent of branching explored. While additional branches often contain relevant contextual cues embedded in sibling or cousin nodes, incorporating them can also reduce the likelihood of getting trapped in local cycles or shallow regions of the graph, leading in many cases to deeper, more informative walks. Furthermore, current graph-based sampling techniques typically base their transitions on the semantic relevance to the last visited node^2^, rather than prioritizing semantic relevance to the target utterance. This often results in the omission of key signals necessary for accurate interpretation. To address these issues, we advocate for models that reason across broader structural dimensions while preserving semantic relevance to the target utterance.

This work addresses the role of discourse structure in online conversations and demonstrates its importance for classification tasks by showing that incorporating it improves the performance of machine learning models across tasks like hate speech detection and polarity prediction. We argue that discourse in online forums follows a two-dimensional structure, where relevant context may arise not only from parent or ancestor comments but also from sibling or cousin nodes. To model this, we introduce the 2D Similarity-based Random Walk, a novel context sampling method that explores multiple paths simultaneously through the discussion graph by allowing expansion from any visited node (*i.e.*, multiple heads). These paths are guided not only by structural connections but also by semantic similarity to the target utterance, allowing the sampling process to focus on the most relevant utterances. Importantly, the proposed method operates at the data level by constraining and selecting contextual utterances used for training and fine-tuning, rather than modifying model architectures or learning objectives.

**Fig. 1:**
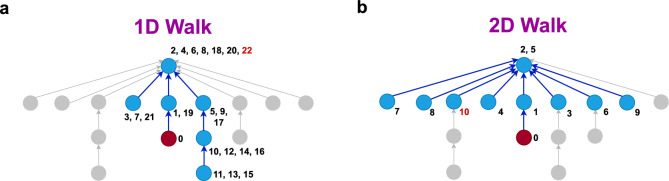
An illustration of (**a**) 1D and (**b**) 2D similarity-based random walks, both executed on the same utterance from the Guest dataset. The start node $v_0$ is shown in red. Nodes and edges visited by the walk are shown in blue, while unvisited nodes and edges are shown in gray. Children nodes point to their parent node (the utterance they reply to). The numbers next to each node denote the visiting time of the walk. The number shown in red color denotes the point at which the walk terminates. Each walk is configured with a maximum length of $L = 10$. While the 2D-walk returns 10 distinct utterances, the 1D-walk terminates prematurely after consecutively revisiting *L* previously visited nodes, yielding only 7 utterances. The 2D-walk explores multiple paths simultaneously, reducing the likelihood of repeatedly visiting the same nodes, and resulting in coverage of more branches and greater diversity in traversal compared to the more linear 1D-walk. Additionally, we note that many conversation trees in the real world are quite small and/or shallow. As a result, a 1D random walk quickly reaches the end of the path and cannot explore much of the conversation. In contrast, the 2D-walk method enables exploration across multiple branches simultaneously, allowing it to cover a larger and more comprehensive portion of the conversation.

We contrast this approach with the 1D Similarity-based Random Walk introduced in prior work, which follows a sequential, single-path traversal by expanding only the most recently visited node (i.e., one head)^2^. We show through empirical analysis that the 2D-walk captures a broader and potentially deeper contextual view and produces more utterances per sample. This leads to measurable gains in classification performance. We evaluate our method on two benchmark datasets: Guest^11^ for hate speech detection, and Kialo^14,15^ for polarity prediction in structured debates. Across both tasks, and using multiple state-of-the-art models including GPT-4^18^, Multi-Head Attention^2^, and BERT^19^, the 2D-walk consistently outperforms both the 1D baseline and the 0D (no-context) approach, highlighting the importance of discourse-aware context modeling. Additional experiments (see Supplementary Methods) show that the gains of the 2D-walk are not solely due to increased sample size. Also, comparisons with an unstructured semantic similarity baseline indicate that structured traversal with semantic guidance can capture context more effectively. The key contributions of this paper are as follows.

- We propose the 2D Similarity-based Random Walk, a novel context sampling algorithm that explores multiple paths simultaneously in discourse graphs to capture richer contextual information.
- We show through empirical analysis that the proposed 2D-walk covers a wider range of branches in the discourse while maintaining the same asymptotic time complexity, compared to the 1D-walk proposed in the literature^2^.
- Our empirical analysis shows that the 2D-walk generates more utterances per sample compared to the 1D-walk, indicating its comprehensiveness.
- We apply our approach to two benchmark datasets: Guest for hate speech detection and Kialo for polarity prediction, and demonstrate its effectiveness across both domains.
- Comprehensive experiments across a range of models, including GPT-4^18^, Multi-Head Attention^2^, and a standard BERT model^19^ with a softmax classification head, confirm that the 2D-walk strategy consistently outperforms both traditional single-path (1D) and no-context (0D) baselines in discourse-based classification.
- Additional experiments controlling for sample size and comparing against a random sampling baseline demonstrate that the observed gains are not solely driven by the number of sampled utterances.
- Comparisons with an exhaustive semantic similarity-based baseline (unstructured) show that semantic similarity alone is insufficient in many cases; by combining structured traversal with semantic guidance, the 2D-walk captures complementary contextual information while remaining computationally efficient.

In addition, we conduct extensive ablation and robustness analyses including uncertainty-aware GPT-4 prompting with abstention, retry-budget sensitivity, and walk-length analysis to better understand the behavior of the proposed method. This paper is structured as follows. The “Background and related work” section discusses context modeling in conversational classification, graph-based approaches, similarity-guided random-walk sampling and classical random-walk variants, with applications in hate speech detection and polarity prediction. The “Methods” section describes the datasets used for analysis and evaluation, and details the sampling algorithms (1D and 2D). The “Empirical analysis” section presents an empirical analysis comparing the structure and content of samples generated by the two walks, including depth, breadth, semantic relevance, and utterance count. The “Classification performance” section evaluates performance across multiple models and datasets, demonstrating the advantages of the 2D approach compared to several baselines, while analyzing its computational efficiency. The Supplementary Information includes additional analysis on methodological robustness, model uncertainty, context ordering, sample size, and retry budget. Finally, the “conclusion” section summarizes the paper and highlights its main contributions.

## Background and related work

### Context modeling in conversational classification

Many text classification tasks in online discussions—such as hate speech detection, stance identification, and polarity prediction–require access to conversational context beyond a single utterance. Early approaches typically incorporated context by concatenating surrounding turns or appending previous utterances as additional input tokens^20^. While simple, such strategies often fail to capture long-range dependencies, focus shifts, and implicit references (e.g., coreference or ellipsis) that naturally arise in threaded or multi-turn conversations^21^.

To address these limitations, recent work has increasingly modeled conversations as structured objects, most commonly graphs, where nodes represent utterances (or words) and edges encode conversational relations such as reply-to links, temporal order, or speaker interactions. This formulation enables more flexible context aggregation and supports reasoning over non-sequential conversational structures.

### Graph-based conversation modeling

Graph neural networks (GNNs) have become a prominent tool for exploiting conversational structure. GraphFlow^21^ constructs a context graph at each conversation turn, where nodes represent words and edges encode relationships based on the current utterance and previous conversation turns. The context is represented in this dynamically constructed graph, and a recurrent GNN propagates information across nodes, enabling the model to learn dependencies from both the current utterance and the conversation history. Building on this line of work, subsequent research such as GraphFlow+^22^ further explores alternative graph construction strategies and recurrent GNN architectures to more richly encode semantic relationships and conversational dynamics.

In the domain of emotion recognition in conversation, the work by Ghosal et al.^23^ introduces DialogueGCN, a graph convolutional neural network that constructs a graph where nodes represent utterances and edges encode sequential and speaker-level dependencies. Context is obtained by connecting each utterance to relevant neighbors based on adjacency and speaker interactions, and this graph is then processed by graph convolution layers to propagate information across the entire conversation. Variants and extensions of graph-based emotion recognition models that build on these ideas include hierarchical and multimodal graph neural architectures that incorporate additional context and modalities to improve utterance-level classification accuracy (e.g.,^24,25^). These works illustrate how GNNs leverage the entire conversational structure to capture multi-hop dependencies, enabling richer contextual understanding for classification tasks.

### Similarity-based random walks for context sampling

An alternative and complementary approach to full-graph message passing is to *sample* conversational context prior to model training and inference using random walks guided by semantic similarity. In this setting, similarity-based random walks are not used as part of the learning architecture itself, but rather as a preprocessing and context selection mechanism that determines which conversational utterances are provided as input to downstream models^2^.

A similarity-based random walk starts from a target utterance node and probabilistically traverses neighboring nodes in the conversation graph according to their semantic similarity. The sampled nodes collectively form a context subset that is subsequently fed into representation learning or sequence modeling architectures, such as SentenceTransformer-based encoders, multi-head attention models, or LLMs, where they influence both training and inference outcomes.

Concretely, given a target node, similarity scores between the target and its neighbors are computed using a text similarity function–commonly cosine similarity over Sentence-BERT embeddings–which has been widely adopted in semantic matching and retrieval tasks^26–28^. These similarity scores are normalized to form a probability distribution, from which the next node is sampled. The walk may ignore edge direction, allowing traversal across parent, child, and sibling replies within a discussion tree. As a result, the sampled context is both structurally flexible and semantically relevant.

Unlike graph neural message passing, similarity-based random walks do not propagate or transform node representations within the graph. Instead, they act as a semantic-aware sampling strategy that selects a limited but informative subset of the conversation. This distinction is particularly important in conversational classification tasks, where providing overly large or weakly related context to downstream encoders can degrade performance. While Sentence-BERT is commonly used to compute similarity scores, the sampling framework is agnostic to the choice of embedding model or similarity metric^15^.

From this perspective, similarity-guided walks provide a lightweight alternative to full graph propagation by decoupling context selection from model architecture. Rather than learning how to aggregate all neighboring nodes, the model receives a curated, semantically relevant context subset, allowing standard encoders to focus on informative conversational signals. This makes similarity-based random walks particularly suitable when the goal is to improve downstream classification performance through controlled, relevance-driven context augmentation rather than explicit graph representation learning.

### Relation to classical random-walk variants

Similarity-based random walks can be viewed as random walks on weighted graphs, where edge weights correspond to semantic similarity between utterances. From this perspective, our approach is closely related to several well-established variants in the random-walk literature, while differing in both objective and operational role.

#### Random walks with teleportation (PageRank-style)

Random walks with teleportation, such as PageRank, assign transition probabilities based on the structure and quality of connections in a graph. In PageRank, links act as weighted votes: transitions are more likely toward pages that receive many or high-quality incoming links, and a teleportation step allows the walker to jump to any page, ensuring global exploration and preventing the walk from getting trapped in local regions^29^. The resulting stationary distribution reflects the global importance or authority of nodes in the network. Our 2D similarity-based random walk follows the same general principle of probabilistic transitions on a weighted graph, but the weights are defined by semantic relevance rather than structural authority. Instead of link quality or connectivity, transition probabilities are computed from semantic similarity between utterances, encouraging the walk to move toward content that is most relevant to the classification target. The proposed walk further differs in how it maintains relevance over time. While PageRank relies on teleportation to uniformly reintroduce global exploration, the 2D-walk preserves and expands a candidate set and repeatedly scores all candidates against the original utterance. This keeps the walk anchored to the task-specific semantic goal, enabling broad yet focused exploration without relying on uniform random jumps.

#### Random walks with stochastic resetting

Random walks with stochastic resetting periodically return the walker to a designated node, which has been shown to improve search efficiency and reduce mean first passage times on complex networks^30^. Resetting alters the stationary distribution and mitigates the risk of the walker drifting far from regions of interest. While our method does not explicitly incorporate a resetting probability, the proposed walk plays an analogous role: by conditioning transitions on the target utterance across multiple candidate paths, the walk repeatedly re-centers exploration around the target. This implicit form of resetting reduces the likelihood of semantic drift and alleviates the tendency of single-path walks to become trapped in locally coherent but task-irrelevant regions of the conversation graph.

#### Self-avoiding walks

Self-avoiding walks explicitly prohibit revisiting previously visited nodes and have been widely studied as models of constrained exploration in statistical physics and network theory^31,32^. While this encourages coverage of new regions of the graph, it can be overly restrictive in finite or sparsely connected conversational structures. In contrast, our proposed walk allows node revisitation and does not impose hard avoidance constraints. Redundancy is mitigated through stochastic exploration and candidate expansion, which reduces the likelihood of repeatedly selecting the same nodes while still permitting revisits when they serve as gateways to previously unvisited but relevant utterances. This flexibility is particularly important in conversational graphs, where revisiting key utterances can enable access to additional informative context through alternative paths.

Overall, while drawing inspiration from these classical random-walk variants, our approach differs in its primary purpose. Similarity-based random walks are employed not as a standalone stochastic process for ranking, search, or graph analysis, but as a semantic-aware context sampling mechanism that constructs informative input for downstream neural classifiers. The proposed 2D formulation further extends this idea by aggregating evidence across multiple candidate paths, improving robustness and contextual coverage without introducing additional model complexity.

### Applications in hate speech and polarity prediction

Hate speech detection has been extensively studied across a variety of paradigms. Early work utilized traditional machine learning models and shallow text features^33,34^, but recent advances have seen a shift toward deep learning and transformer-based architectures, which consistently yield state-of-the-art performance^4,35,36^. Transformer models like BERT and its variants have been particularly effective, often enhanced through fine-tuning and domain-specific adaptations^37,38^. Multimodal approaches have also emerged, integrating audio and text modalities for richer semantic capture in hate detection tasks^39^.

Polarity prediction has similarly evolved from traditional methods such as rule-based and lexicon-based scoring^40,41^ to more context-aware approaches leveraging contextual embeddings and transformer models^42,43^. Graph-based models have been introduced to capture the structural and contextual nuances of conversations. Notably, GraphNLI employs graph walk techniques to incorporate wider conversational context, enhancing the prediction of argumentative relations between posts^15^. By performing root-seeking random walks, GraphNLI captures surrounding context, leading to improved performance over baseline models in the polarity prediction task.

While random-walk models have been explored for feature weighting and representation learning in text classification^44,45^, their application as sampling strategies for constructing training data in transformer-based architectures is less prevalent. Traditional random-walk methods, such as DeepWalk^46^, focus on learning node representations through sequences generated by random walks, primarily for tasks like node classification and link prediction. However, the integration of similarity-based random-walk sampling strategies to enhance contextual relevance and diversity in training data for discourse classification remains underexplored.

Recent advancements in contextual sampling for discourse classification include a transformer-based model that uses a 1D similarity-based random walk to sample context from online discussions^2^. While effective, this approach is limited by single-path traversal and guides transitions based on semantic similarity to the last visited node rather than the target utterance. In contrast, our method explores multiple paths simultaneously and centers transitions around the target utterance, capturing broader and more relevant discourse context for classification.

## Methods

### Datasets description

#### Guest dataset

The first dataset used in this study was curated by Guest et al.^11^, which focuses on detecting hate speech in online platforms. The content originates from Reddit and specifically targets one form of gender-based hostility: *misogyny*. Each utterance has been manually labeled by experts, with labels distinguishing between misogynistic and non-misogynistic utterances.

The dataset is organized into 604 discussion trees, where each tree begins with a root node representing an initial post or a start of a conversation, followed by a sequence of nodes representing replies. Each reply in the tree is connected to one parent node (the utterance it is replying to), while potentially having multiple children nodes (the replies to it). This hierarchical structure enables us to model contextual interactions between users. For our sampling strategy, we perform both 1D and 2D random walks through these trees. Each sample starts with the target utterance—the one to be classified—followed by up to $L-1$ additional utterances collected using the walk—representing the context of the first utterance—, where *L* is the walk length. We omit samples with only one utterance (i.e., a node that has no parent or children), as they lack surrounding context. This dataset is imbalanced, with significantly fewer misogynistic instances. The distribution is as follows:

- Misogynistic comments: 515 samples (8.08%)
- Non-misogynistic comments: 5861 samples (91.92%)

#### Kialo dataset

For the polarity prediction task, we use the Kialo dataset^15^, which consists of structured online debates collected from the Kialo platform^14^, a website designed for collaborative argumentation and decision-making. Debates are initiated when a user posts a central claim, known as the thesis. Other participants contribute by replying with statements that either support or attack the utterances they respond to. These interactions naturally form a tree structure, where the root node corresponds to the thesis, and every subsequent node is a reply to exactly one parent.

The dataset includes 1560 full discussion threads, each capturing the hierarchical flow of arguments and the logical relationships between them. Every node (except the root) in the tree represents a reply and is annotated as either supporting or attacking the parent node. In addition to the reply structure and argument texts, the dataset includes metadata such as timestamps, author metadata, and user votes on the perceived impact of each argument. Unlike open forums, Kialo enforces moderation standards that result in well-formed, standalone argumentative units, making the dataset particularly suitable for structured discourse analysis.

To prepare samples for classification, we run the 1D and 2D random walks, as described in the previous section. Each sample begins with a target utterance and includes up to $L - 1$ other utterances (context) from the same discussion tree, selected through either 1D or 2D traversal. We retain only samples that have more than one utterance, ensuring each sample has contextual data.

The dataset has a relatively balanced class distribution, with the breakdown as follows:

- Attacking comments: 186,316 samples (52.62%)
- Supporting comments: 167,740 samples (47.38%)

#### Datasets and contextual challenges

We selected the Guest and Kialo datasets for this study because they present challenging classification problems that heavily depend on contextual information^2,15^. These tasks are challenging because, in many cases, the correct label of a comment or argument cannot be determined from the text alone, and understanding the surrounding discussion is crucial^13^. For instance, in the Guest dataset, a comment may appear benign or sarcastic in isolation but becomes misogynistic when interpreted within its conversational context^11^. Similarly, in Kialo, whether an argument supports or attacks the parent statement depends on the logical structure and discourse flow of the debate, rather than solely on the lexical content of the reply^15^.

Both datasets are naturally graph-like, with hierarchical structures that explicitly encode reply-to relations between utterances, making them well suited for context modeling via random walks and graph-based traversal. Moreover, they represent fundamentally different yet important tasks: Guest focuses on hate speech detection in noisy, informal social media conversations, while Kialo addresses polarity prediction in moderated, structured argumentative discourse.

Beyond their contextual complexity, we also chose these datasets due to their high annotation quality and reliability. The Guest dataset was annotated by trained expert annotators following a carefully designed taxonomy and detailed annotation guidelines, addressing known limitations of crowdsourced labeling for sensitive phenomena such as misogyny^11^. In Kialo discussions, the stance of a comment is explicitly specified by its creator and supported by the platform’s structured debate format^14^. The combination of expert-driven and platform-supported annotations provides high-quality supervision signals across two distinct discourse settings, enabling a robust evaluation of context-aware classification models.

### 1D and 2D similarity-based random walks

The 1D and 2D Similarity-based Random Walks are tailored for navigating conversational structures in directed graphs, where each node represents an utterance and edges represent conversational turns or reply-to relations. These methods combine stochastic exploration with cosine similarity to guide walk transitions toward semantically relevant utterances.

Let the discussion graph be denoted as $G = (V, E)$, where:

- $V$ is the set of utterance nodes,
- $E \subseteq V \times V$ is the set of directed edges,
- $v_0 \in V$ is the starting node (target node representing the utterance to be classified),
- $L \in \mathbb {N}^{+}$ is the walk length, i.e., the number of nodes (utterances) to include,
- $M \in \mathbb {N}^{+}$ is the maximum number of retries allowed if a transition leads to an already visited node. If the number of retries exceeds *M*, the walk terminates.

Since transitions are guided by semantic similarity, a walk may occasionally return to an already visited node, particularly in shallow branches, dense graphs, or when semantically similar utterances dominate the local structure. This is further exacerbated when the number of distinct nodes is smaller than the desired walk length *L*, making revisits inevitable. When the walk transitions to a node that has already been visited, we allow the transition but do not include the repeated node’s utterance in the final sample, as it has already contributed context. Instead, we attempt to retry the step by selecting an alternative neighbor. This mechanism prevents the walk from including redundant context.

To prevent infinite loops or inefficient backtracking, especially in shallow or exhausted subgraphs, we introduce a retry budget *M*. If the walk fails to find a valid (unvisited) node after $M+1$ retries, the walk terminates early. This design ensures the algorithms remain efficient and avoid degenerate cases where the walk continues pointlessly. For example, in a walk traversing a small conversation tree, it is possible that all nodes have already been visited. After $M+1$ such failed attempts, we conclude that no further useful context remains, and we stop the walk.

In our experiments, we set $M = L$ for both algorithms to maintain a reasonable bound on complexity. This setting also ensures a fair comparison between the two algorithms, as they are both constrained by the same upper limit on the number of steps, thereby isolating the effect of their structural differences rather than confounding it with varying capacities. In the section “Retry budget analysis” of the Supplementary Information, we show that setting $M = L$ for our experiments provides an effective point where the 2D-walk achieves its highest observed performance on the downstream task.

#### 1D similarity-based random walk

This walk, which was proposed by Agarwal et al. ^2^, starts from the utterance $v_0$, appends it and its parent (if available) to the walk sequence, and then performs $L - 2$ additional transitions (iteratively). The inclusion of the parent node in the walk is prioritized because it provides essential contextual grounding. At each step, the algorithm collects the current node’s parent and children as candidates (choices). The next node is then selected randomly from the list of candidates, with probability proportional to the cosine similarity between each candidate and the current node, computed using a Sentence-BERT (S-BERT) encoder^47^. This localized (1D) similarity focus ensures that transitions favor semantically adjacent utterances, leading to a sequential walk, centered around the most recent context.

#### 2D similarity-based random walk

The variation we propose also begins at $v_0$, initializing the walk in a similar manner. However, unlike the original approach, the candidate list is not reset at each step. Instead, it is preserved and expanded with new neighbors of the currently selected node, allowing the algorithm to explore multiple paths simultaneously. Additionally, at each iteration, rather than comparing the current node with its immediate neighbors, the algorithm computes similarity scores between the original utterance $v_0$ and all candidates in the list to select the most relevant one. This global (2D) similarity strategy keeps the walk anchored to the classification goal, promoting the selection of utterances that remain contextually aligned with $v_0$ across the entire trajectory. Additionally, exploring multiple paths enables a richer and more robust discovery process, potentially capturing diverse but semantically consistent utterances that support improved classification performance.

We note that unlike the 1D-walk, which encodes discursive distance through linear position, the 2D-walk adopts a graph-based notion of context. While traversal may move across different branches of the discussion tree, all sampled utterances remain structurally connected to the target utterance and are selected based on similarity to it, ensuring semantic alignment throughout the walk.

Importantly, the 2D-walk does not allow jumping to arbitrary nodes. At each step, selection is restricted to the candidate list, which contains only parents or children of previously visited nodes that have not yet been expanded. Thus, a node becomes eligible only if it is directly adjacent in the conversation tree to an already visited node. Consequently, every newly added node is exactly one edge away from the current walk, and the sampled context always forms a connected subgraph rooted at $v_0$, ensuring incremental expansion rather than long-range jumps. An alternative way to view the procedure is as a walk that expands simultaneously along multiple active paths (or ”heads”), while remaining fully connected and never teleporting to previously visited nodes.

The pseudocode for both algorithms is provided in Algorithms 1 and 2. The key differences introduced in the 2D-walk compared to the 1D-walk are underlined in Algorithm 2. Additionally, Fig. 1 presents a visual example of 1D and 2D walks executed on the same utterance (shown in red), illustrating the advantages of the 2D-walk’s simultaneous exploration of multiple paths. Compared to the 1D-walk, the 2D-walk achieves broader coverage and retrieves a larger number of utterances.

**Algorithm 1:**
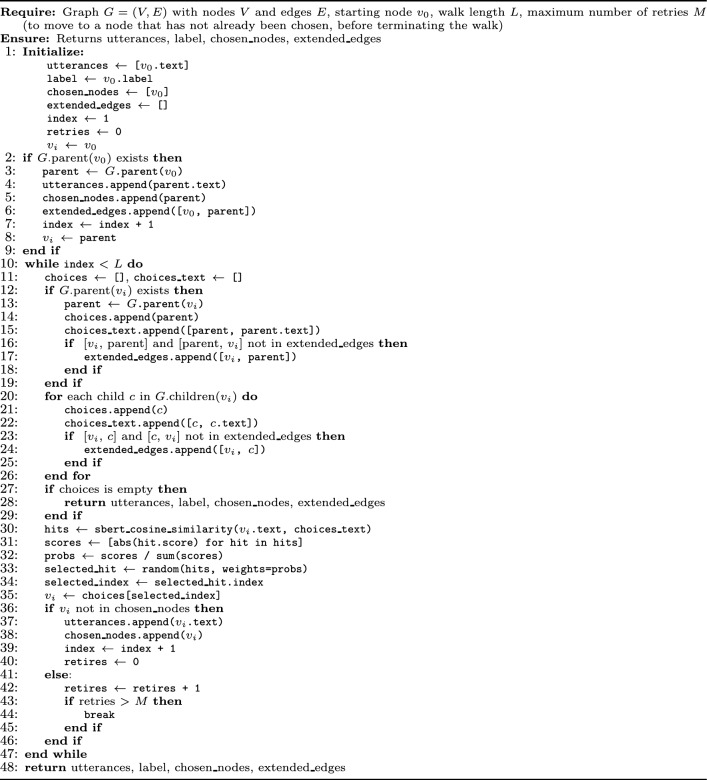
1D similarity-based random walk.

#### Time complexity analysis

Both the 1D and 2D Similarity-based Random Walk algorithms have an upper-bound time complexity that depends on the walk length $L$, the node degree $d$, and the cost of computing semantic similarity between text pairs using S-BERT, denoted $C$.

**Algorithm 2:**
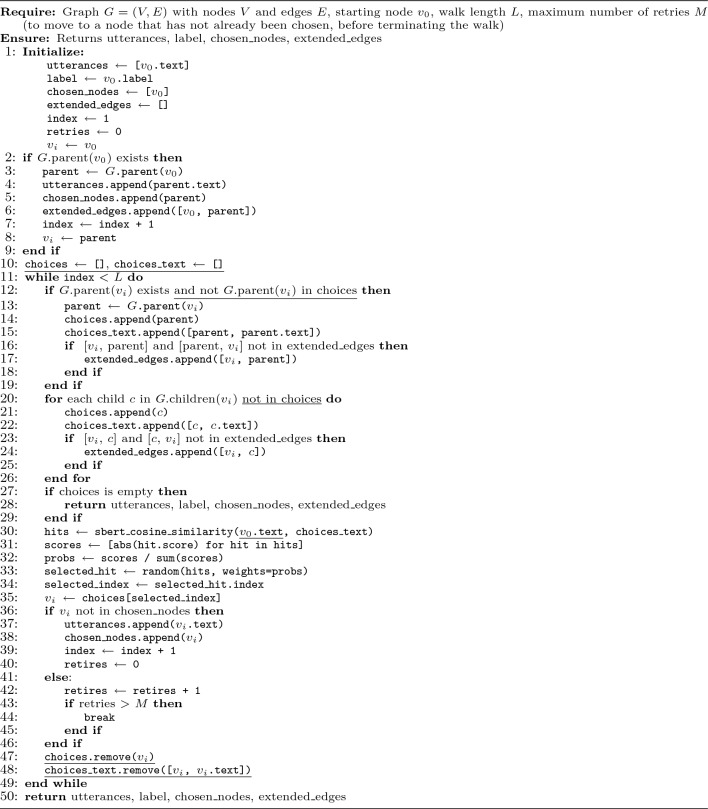
2D similarity-based random walk.

At each of the $L$ steps, the 1D-walk evaluates all neighbors of the current node–i.e., up to $d$ candidates, where $d$ is the node’s degree. In the worst case, this can be as high as $d_{\max }$ across the graph. However, to avoid revisiting nodes, the algorithm may retry multiple times. In our setting, the number of retries is capped at $M = L$, resulting in up to $L$ retries per step. This yields a worst-case time complexity of:

1 $$\begin{aligned} \mathcal {O}(L^2 \cdot d_{\max } \cdot C) \end{aligned}$$

This upper bound captures the case where the walk repeatedly encounters already visited nodes at every step. While unlikely in sparse or well-connected graphs, it was included for completeness.

An important difference in the 2D-walk is that the candidate pool is expanded globally across steps. Therefore, at step $t$, the pool may contain up to $t \cdot d_{\max }$ nodes. The algorithm also performs retries when revisiting nodes, up to $L$ times per step. Thus, each retry at step $t$ can involve comparing against up to $t \cdot d_{\max }$ candidates. Aggregating over all $L$ steps, the worst-case total number of similarity evaluations becomes:

$$\begin{aligned} \sum _{t=1}^{L} L \cdot t \cdot d_{\max } = L \cdot d_{\max } \cdot \sum _{t=1}^{L} t = L \cdot d_{\max } \cdot \frac{L(L+1)}{2} = \mathcal {O}(L^3 \cdot d_{\max }) \end{aligned}$$

This leads to a worst-case time complexity of:

2 $$\begin{aligned} \mathcal {O}(L^3 \cdot d_{\max } \cdot C) \end{aligned}$$

The cubic term arises from the combination of retries, expanding candidate pool, and the number of steps. Although this is a pessimistic bound and unlikely in most real-world cases, it accurately reflects the theoretical upper limit. We also note that while nodes selected during primary steps as well as retries are removed from the candidate pool, resulting in at most $L^2$ total removals across the walk, this only reduces the total number of similarity computations by a lower-order term and does not change the asymptotic complexity.

In our experiments, the walk length $L$ is set to a small constant (*i.e.,* $L = 10$). This is consistent with prior work in related domains where values like $L = 6$ are commonly used^2,15^. As a result, when $L$ is treated as a constant—as is standard in many NLP applications—the asymptotic time complexity of both 1D and 2D walks is equivalent and reduces to:

3 $$\begin{aligned} \mathcal {O}(d_{\max } \cdot C) \end{aligned}$$

## Empirical analysis

### Graph statistics

**Table 1:** Structural statistics of discourse graphs in the Guest and Kialo datasets.

Statistic	Guest	Kialo
Mean number of nodes per graph	10.57	202.55
Mean number of edges per graph	9.57	201.55
Mean depth	2.98	5.89
Maximum depth	10	18
Mean breadth	2.65	2.60
Maximum breadth	16	224

Table 1 summarizes the structural properties of discourse graphs in the Guest and Kialo datasets prior to applying random walks. Guest discussion graphs are relatively compact, with an average of approximately 10.6 nodes and 9.6 edges per graph. The mean depth is 3.0, with a maximum observed depth of 10, indicating that most discussions are shallow, with only a small number exhibiting longer conversational chains. The mean breadth is 2.65, with a maximum breadth of 16, reflecting occasional high branching.

In contrast, Kialo graphs are substantially larger and more complex, containing on average over 200 nodes and edges per discussion. The mean graph depth increases to 5.89, with a maximum depth of 18, indicating the presence of deeper argumentative structures. While the mean breadth remains comparable to Guest (2.60), the maximum breadth reaches 224, highlighting the existence of highly branched discussion points in some debates. These statistics demonstrate that Kialo exhibits both greater scale and greater structural variability, and they provide natural upper bounds that can be used to contextualize random-walk-based depth and breadth measurements across datasets.

### Walk structure—depth and breadth

To better understand the behavior of the proposed 2D-walk compared to the 1D-walk, we analyzed the structure of the generated walks along two dimensions: *depth* and *breadth*. These measures capture how far and how widely a walk explores the graph from the starting utterance.

We define the *depth* of the walk as the length of the longest simple path starting from the initial node $v_0$ (*i.e.,* the node corresponding to the utterance to be classified)^48^. A simple path is a sequence of distinct nodes where each consecutive pair is connected by a directed edge in the walk. Let $E' \subseteq E$ be the set of directed edges traversed during the walk. Then the depth $D$ is given by:

4 $$\begin{aligned} D = \max \left\{ \, |\pi | - 1 \;\vert |\; \pi = (v_0, v_1, \dots , v_k),\; (v_i, v_{i+1}) \in E',\; v_i \ne v_j \;\forall i \ne j \, \right\} \end{aligned}$$

where $|\pi |$ is the number of nodes in the simple path $\pi$.

Justification: Since the goal is to classify an utterance at the starting node $v_0$, we focus on the structure of the walk that extends outward from this node. Measuring the longest simple path starting from $v_0$ captures how far the walk can explore from the current utterance without revisiting any node, thereby reflecting the potential depth of contextual or argumentative information considered in the walk.

We define the *breadth* of the walk as the average number of outgoing edges among the nodes that have at least one outgoing edge in the walk^49^. Let $V' \subseteq V$ be the subset of nodes visited by the walk, and let $E' \subseteq E$ be the set of directed edges traversed during the walk. Define the set of branching nodes as:

$$\begin{aligned} P = \left\{ u \in V' \;\vert |\; \exists v \in V' \text { such that } (u, v) \in E' \right\} \end{aligned}$$

Then the breadth $B$ is given by:

5 $$\begin{aligned} B = \frac{1}{|P|} \sum _{u \in P} \left| \left\{ v \in V' \;\vert |\; (u, v) \in E' \right\} \right| \end{aligned}$$

Justification: In Eq. (5), we include only the nodes with at least one outgoing edge (i.e., nodes in $P$) in the average to meaningfully capture how branchy or exploratory the walk is. Nodes with no outgoing edges contribute nothing to the walk’s branching structure, and including them would dilute the measure. For example, suppose a walk covers five nodes, where only one node has outgoing edges to the remaining four. If we include all nodes, the average branching factor becomes $\frac{4}{5} = 0.8$, which underestimates the actual branching behavior at the decision point. Instead, restricting to the one branching node gives a value of $\frac{4}{1} = 4$, better capturing the local branching structure. This formulation reflects how much a walk expands when it does branch, providing a clearer signal of the walk’s breadth.

#### Walk structure analysis

**Fig. 2:**
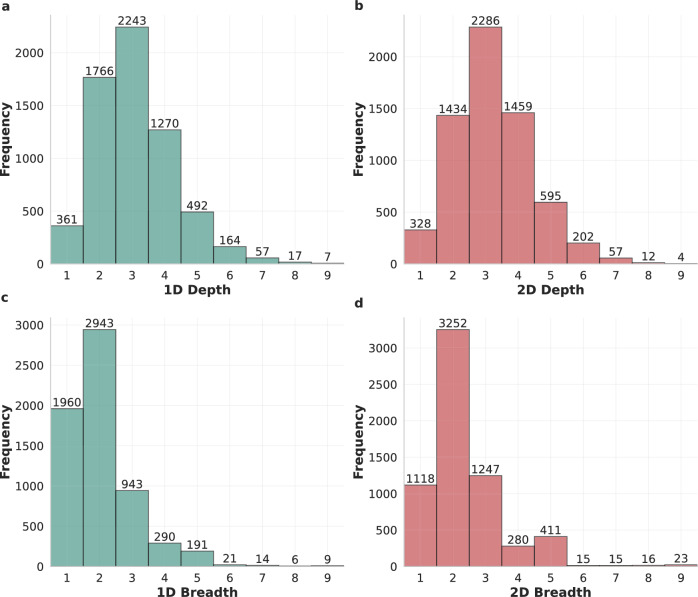
Depth and breadth distributions of 1D and 2D Similarity-based Random Walks in the Guest dataset. (**a**,**b**) The depth histograms for 1D and 2D walks, respectively, while (**c**,**d**) show the corresponding breadth histograms. Frequencies indicate the number of walks observed at each depth or breadth level. The histograms illustrate that 2D-walks achieve greater depth and breadth in this dataset compared to 1D-walks.

**Fig. 3:**
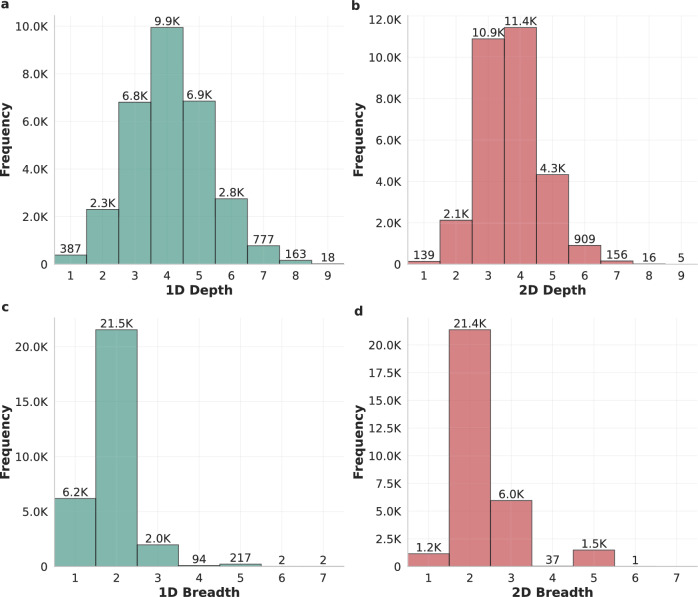
Depth and breadth distributions of 1D and 2D similarity-based random walks in the Kialo dataset. (**a**,**b**) The depth histograms for 1D and 2D walks, respectively, while (**c**,**d**) show the corresponding breadth histograms. Frequencies indicate the number of walks observed at each depth or breadth level. The histograms illustrate that 2D-walks achieve lower depth in this dataset but greater breadth compared to 1D-walks.

We computed the depth and breadth of thousands of 2D-walks and their corresponding 1D-walks, where each pair was executed on the same target utterance to ensure a fair comparison. This was done over the two datasets: Guest and Kialo. For the Guest dataset, we sampled 6,376 walks, while for the Kialo dataset we analyzed a sample of 30,000 walks. The results are presented in Figs. 2 and  3, which show the distributions for both datasets.

Figure 2 presents the depth and breadth distributions for 1D and 2D walks on the Guest dataset. A paired *t*-test at the walk level indicates that 2D-walks achieve greater depth ($t = -8.61$, $p = 9.33 \times 10^{-18}$) and greater breadth ($t = -30.96$, $p = 3.71 \times 10^{-196}$) than their corresponding 1D-walks. The magnitude and consistency of these differences across a large number of paired observations explain the extremely small *p*-values.

Figure 3 shows the corresponding distributions for the Kialo dataset. In contrast to Guest, 1D-walks reach greater depth on average than 2D-walks ($t = 47.73$, $p \approx 0$), while 2D-walks exhibit markedly higher breadth ($t = -114.07$, $p \approx 0$). This reversal in depth reflects a dataset-specific structural effect rather than a contradiction of the general behavior of the methods. As discussed below, the dense and richly connected Kialo discussion graphs, along with their greater depth compared to Guest (Table 1), reduce the frequency of premature termination in 1D-walks, allowing these walks to sustain long single-path traversals, hence, reaching a greater depth on average compared to 2D-walks. In both datasets, however, the breadth advantage of 2D-walks is large and consistent.

#### Relative depth and breadth analysis

**Fig. 4:**
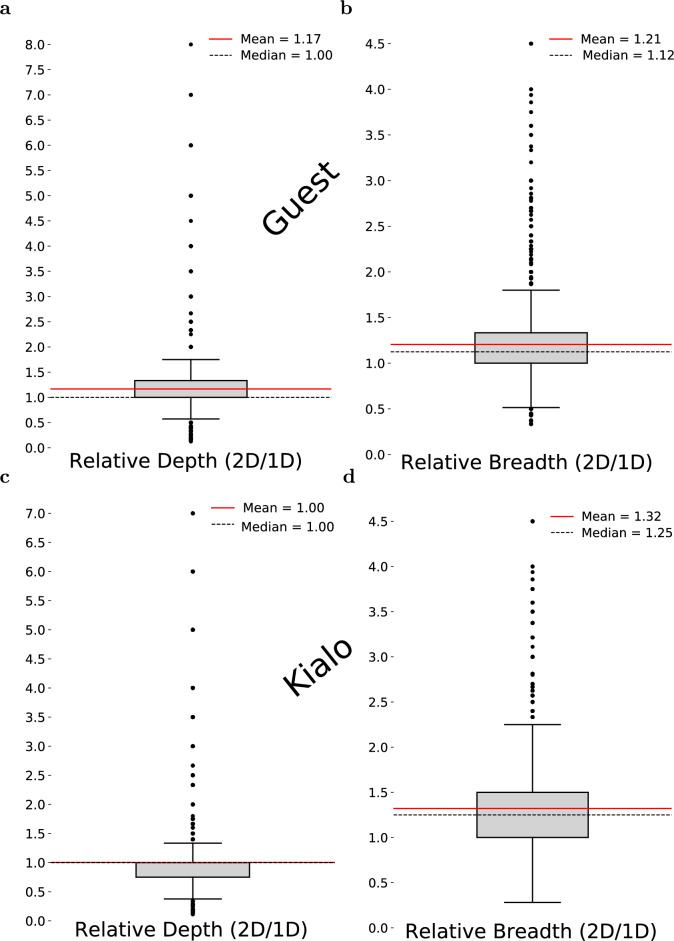
Distribution of relative depth and breadth for 2D vs. 1D similarity-based random walks. (**a**) Relative depth on Guest dataset. (**b**) Relative breadth on Guest dataset. (**c**) Relative depth on Kialo dataset. (**d**) Relative breadth on Kialo dataset. The relative depth and breadth are defined as the ratio between the depth/breadth of the 2D-walk to that of the 1D-walk. The depth of the walk is defined as the length of the longest simple path (i.e., a path with no repeated nodes) from the starting node. The breadth is defined as the mean out-degree of the nodes that have at least one outgoing edge. Overall, the 2D-walk exhibits greater breadth and comparable or higher depth than the 1D-walk.

To complement the direct comparison of empirical distributions, we also computed relative depth and relative breadth, defined as the ratio between the depth (or breadth) of a 2D-walk and that of its paired 1D-walk. These ratios provide a compact summary of walk-level differences but should be interpreted as descriptive rather than inferential statistics. The distributions are shown in Fig. 4.

On the Guest dataset (Fig. 4a), the relative depth distribution has a mean of 1.17 and a median of 1.00, indicating that 2D-walks typically achieve depth comparable to, and occasionally exceeding, that of 1D-walks. While many ratios cluster near or below 1 (corresponding to cases where 1D-walks are deeper), there exist outlier cases in which 2D-walks extend substantially further. These outliers reflect scenarios in which the 1D-walk terminates early due to repeated revisits, whereas the 2D-walk continues exploring alternative paths. In contrast, relative breadth (Fig. 4b) shows a clear upward shift, with a mean of 1.21 and a median of 1.12, confirming the consistently broader exploration achieved by the 2D strategy.

On the Kialo dataset, the relative depth distribution (Fig. 4c) is centered closer to 1, with substantial mass below 1. This aligns with the histogram-based analysis and confirms that 1D-walks often reach greater depth in these graphs. Importantly, this pattern does not indicate instability of the 2D method but rather reflects the fact that Kialo’s densely connected argumentation graph reduces early termination in 1D-walks. Relative breadth (Fig. 4d), however, again shows a pronounced advantage for 2D-walks, with a mean of 1.32 and a median of 1.25.

Taken together, the histogram-based comparisons establish statistically significant differences in depth and breadth, while the relative ratios provide an intuitive, walk-level summary of how frequently and by how much one strategy exceeds the other.

#### Discussion on walk structure

The observed structural differences between 1D and 2D walks arise from their fundamentally different traversal mechanisms. The 2D-walk maintains and expands a pool of candidate paths at each step, enabling simultaneous exploration of multiple semantically related directions. This design naturally promotes greater breadth, as the walk is less constrained to a single trajectory and can cover a wider region of the discourse graph.

Depth behavior depends more strongly on graph structure. In general, 1D-walks are prone to premature termination when they repeatedly revisit already explored nodes, exhausting the allowed number of retries. The 2D-walk mitigates this failure mode by preserving alternative candidate paths, which reduces the probability of becoming trapped in local cycles and allows the walk to continue progressing. This mechanism explains why 2D-walks tend to achieve greater depth in sparser or less richly connected graphs such as Guest.

In contrast, the Kialo graph contains a large number of densely interconnected utterances organized into extended argumentative chains. This structural richness substantially lowers the risk of early termination for 1D-walks, enabling them to follow long, coherent paths. As a result, 1D-walks often achieve greater depth in Kialo despite the parallel exploration capability of the 2D method. Importantly, even in this setting, 2D-walks consistently achieve higher breadth, reflecting their stronger capacity for lateral exploration across semantically related arguments.

### Semantic similarity

#### Semantic similarity analysis

To better understand the characteristics of the sampled context, we analyzed the average cosine similarity between the target utterance (i.e., the utterance to be classified) and the remaining nodes in the sampled walk. This metric serves as a guiding heuristic for estimating semantic relevance—samples containing more semantically related nodes are more likely to provide useful contextual information for downstream models, although high semantic similarity does not guarantee contextual importance.

For this analysis, we reused the same walks generated in the previous section and computed the mean cosine similarity using S-BERT embeddings for each walk. This was performed over thousands of walks across both the Guest and Kialo datasets. The average similarity scores were then aggregated and plotted to compare the 2D and 1D sampling strategies.

**Fig. 5:**
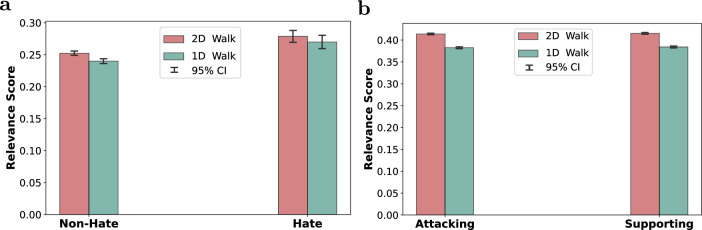
Comparison between 1D and 2D Similarity-based Random Walks in terms of the average cosine similarity between the embeddings of the sampled nodes and the embedding of the target node (i.e., the target utterance) on the (**a**) Guest and (**b**) Kialo datasets. Cosine similarity is computed using utterance embeddings generated by the S-BERT model. Error bars denote 95% confidence intervals computed over all sampled walks. The intervals are generally small, indicating limited variability in semantic similarity across walks, and become extremely narrow (visually collapsed) in the Kialo dataset due to the larger number of samples. The results indicate that the 2D-walk consistently yields samples with higher semantic relevance to the target utterance compared to the 1D-walk.

The results are presented in Fig. 5. As shown, the 2D-walk consistently yields samples with higher semantic relevance to the target utterance than the 1D-walk across all classes and both datasets. This trend holds for both Guest and Kialo. The figure also shows that the 95% confidence intervals are generally small, indicating limited variability in semantic similarity across sampled walks.

#### Discussion on semantic similarity

The key factor contributing to the higher semantic relevance observed in the 2D-walk lies in how similarity is computed. In the 2D-walk, semantic similarity is explicitly measured between the target utterance and each node in the candidate pool during each expansion step. This mechanism ensures that the walk remains anchored to the semantic context of the classification task. In contrast, the 1D-walk operates in a more local manner, where similarity is computed between the current node in the walk and its immediate neighbors, without considering the relevance to the target utterance. This localized strategy can lead to a drift away from the target context, reducing the overall semantic relevance of the sampled nodes.

However, we note that high semantic similarity does not always imply contextual importance, as crucial cues for classification may arise from utterances that are less similar in meaning. Our method preserves discourse structure by constraining sampling to the conversation graph and enforcing mandatory inclusion of the target’s parent utterance. In addition, controlled stochasticity allows occasional exploration beyond highly similar nodes. Together, these design choices ensure that structurally critical but less semantically similar utterances remain accessible during sampling.

Overall, this analysis reinforces the advantage of the 2D-walk in navigating complex conversation structures to retrieve semantically informative context for classification tasks. Its ability to explore multiple paths while maintaining relevance to the target utterance allows it to capture semantically diverse and relevant utterances less likely to be reached via a single linear path.

### Comprehensiveness

#### Analysis of sample size

We recorded the number of utterances per sample generated by the 1D and 2D walks. The goal of this analysis is to determine whether one walk variant tends to produce more utterances per sample than the other. The maximum number of utterances per sample is defined by the walk length $L$, which was set to 10 in our experiments. However, due to early termination—when a walk repeatedly revisits previously seen nodes and exceeds the maximum number of allowed retries–some walks may produce fewer than $L$ utterances. Also, in some cases, the walk terminates naturally when the discussion tree lacks enough nodes to reach the maximum walk length $L$, a situation frequently observed in the Guest dataset.

**Fig. 6:**
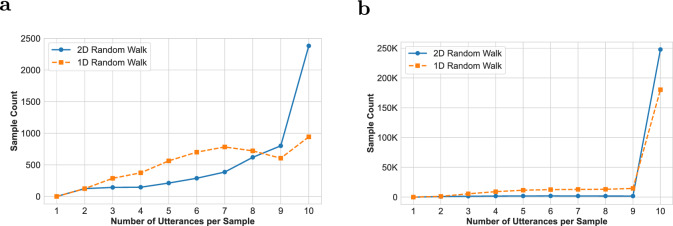
Distribution of the number of utterances per sample for 1D and 2D similarity-based random walks on the (**a**) Guest and (**b**) Kialo datasets. The 2D-walk consistently generates more comprehensive samples—i.e., with utterance counts closer to the maximum walk length *L*=10–compared to the 1D-walk.

We define the comprehensiveness of a walk as the number of utterances it successfully samples, with higher comprehensiveness indicating that the walk reaches or approaches the maximum walk length *L*. We visualized the distribution of utterance counts per sample across both datasets (Guest and Kialo) and walk types (1D and 2D), as shown in Fig. 6.

As illustrated, the 2D-walk consistently generates more comprehensive samples than the 1D-walk. On the Guest dataset (Fig. 6a), the 2D-walk produces the full $L = 10$ utterances in approximately 50% of the samples, whereas the 1D-walk achieves this in only about 20% of the cases. Correspondingly, the average number of utterances per sample is 8.38 for the 2D-walk, compared to 7.01 for the 1D-walk. Most 1D-walk samples are notably shorter than 10 utterances.

On the Kialo dataset (Fig. 6b), approximately 96% of the 2D-walk samples reach the full length of 10 utterances, compared to 69% for the 1D-walk. The average utterance count per sample further reflects this gap: 9.79 for the 2D-walk and 8.89 for the 1D-walk.

#### Discussion on comprehensiveness

The increased comprehensiveness of the 2D-walk samples suggests that this approach is more effective at generating fuller contexts. More comprehensive samples are expected to improve performance in downstream tasks by providing richer contextual information to the model. As explained earlier, the 1D-walk’s sequential exploration increases the likelihood of revisiting the same nodes consecutively (especially when encountering a dead end and backtracking), resulting in early termination. In contrast, the 2D-walk dynamically expands a candidate pool, allowing broader exploration and reducing the risk of local traps, which helps it generate more complete and informative samples.

## Classification performance

Using the Guest and Kialo datasets, we evaluated the classification performance of three transformer-based models: GPT-4, Multi-Head Attention^2^, and a simplified variant we call SoftmaxBERT. The Multi-Head Attention model combines an S-BERT encoder with a multi-head attention mechanism followed by a classification head, whereas SoftmaxBERT attaches a classification head directly to the S-BERT encoder. To assess the impact of contextual sampling, we trained and tested these models on samples obtained using our proposed 2D Similarity-based Random Walk, its 1D variant, a 0D (no-context) variant, as well as two additional baselines. These are Random L, which samples *L* utterances randomly from the conversation graph, and Top L, which selects the *L* utterances most semantically similar to the target, providing an approximate upper bound under the similarity heuristic. The 0D variant includes only the target utterance (or the target and its parent for polarity prediction). This setup allows us to isolate the effect of sampling strategies on classification performance.

### GPT-4

We used the OpenAI package in Python to evaluate the performance of the GPT-4 model on the classification tasks while comparing its performance across the different sampling variants. GPT-4 is a transformer-based LLM developed by OpenAI^18^, built on the decoder architecture of the original Transformer design^50^. It uses stacked self-attention layers, positional embeddings, and layer normalization, enabling it to model long-range dependencies in text. Since GPT-4 is a general-purpose language model capable of classifying text based on a given prompt, we evaluated its performance directly on the test set. To reduce the cost of API usage, we randomly selected 10% of the test set and used this subset to assess the model’s classification performance.

The context obtained through the sampling algorithms consists of the target utterance to be classified along with a set of surrounding utterances that represent the context. However, this context is inherently unordered, as the walk may collect utterances from different branches of the conversation tree without preserving their temporal or reply-to sequence. To make this context more suitable for GPT-4, which benefits from coherent and logically ordered input, we linearized the context using a Depth First Search (DFS) traversal^51^. This step imposes a consistent temporal order on the sampled utterances, helping the language model better understand the flow of conversation and improving classification performance.

**Fig. 7:**
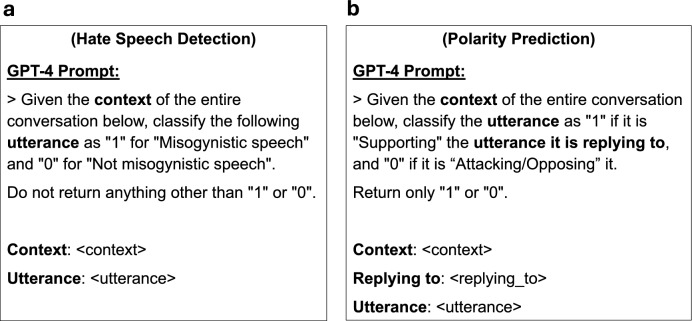
The prompts used with the GPT-4 model to perform the classification tasks: (**a**) hate speech detection and (**b**) polarity prediction. The context is the complete sample obtained using each sampling strategy, while omitted in the case of 0D (no-context) approach. The context is linearized by ordering utterances in temporal order using depth first search (DFS) algorithm. The utterance in the prompt refers to the target comment or post to be classified. For polarity prediction, the parent utterance to which the target is replying is also provided separately in the prompt.

Figure 7 illustrates the prompts used with GPT-4 for the classification tasks of hate speech detection and polarity prediction. The prompt includes the full context obtained from the sampling walk (linearized using DFS traversal), as well as the target utterance presented separately. For polarity prediction, the parent utterance (i.e., the utterance to which the target is replying) is also included as a separate input. We note that in the 0D (no-context) setting, the model receives only the target utterance without any surrounding context.

### Multi-head attention

We trained and tested the Multi-Head Attention model on both datasets (Guest and Kialo). The input to this model consists of the target utterance alongside additional utterances representing the context and obtained by the walk. The embeddings of these utterances are generated using S-BERT, and passed through a multi-head attention module. This attention mechanism uses projections for queries, keys, and values, which are defined through linear layers. Specifically, the parent of the target node (to be classified) attends over its neighborhood nodes via multi-head attention. The aggregated output is then pooled and concatenated with the target node’s embedding and the difference between them. The final concatenated representation is passed through a fully connected classification head. This design allows the model to effectively capture both local textual features and contextual signals from surrounding discussion nodes. In the case of the 0D-walk, where only the target utterance is available, the target node’s embedding is passed through a fully connected layer instead of the multi-head attention layer.

### SoftmaxBERT

To investigate whether our results hold across other models, we trained and tested a simpler variant of the Multi-Head Attention architecture, which we call SoftmaxBERT. This model omits the multi-head attention structure and instead focuses on the utterance embeddings for the classification task. It uses the S-BERT model to encode the input utterances. Then, the embeddings of the context utterances are mean-pooled, and concatenated with the target node’s embedding and their absolute difference. This final representation is then passed through a softmax classification layer to compute the label prediction. While structurally simpler than the Multi-Head Attention model, SoftmaxBERT still benefits from powerful pretrained language representations and serves as a strong baseline to evaluate the general effectiveness of the proposed sampling strategy. We note that in the case of the 0D-walk, only the embedding of the target utterance is passed through the classification layer.

### Experiment details

For the Multi-Head Attention and SoftmaxBERT models, we used an 80/20 train-test split and averaged results over three independent runs, each with a different train-test fold, to ensure robustness. Similarly, for the GPT-4 model, we performed evaluation across three folds to maintain consistency. The same target utterances, model configurations, and hyperparameters were used across all sampling conditions.

The models were optimized using Adam with the default learning rate of 2e-5, which is the standard initial value for transformer models in SentenceTransformers. To improve convergence stability, we applied a linear learning rate warm-up over the first 10% of training steps. This warm-up phase gradually increases the learning rate from 0 to its peak value (2e-5), after which it follows a linear decay schedule. This approach helps avoid large weight updates at the beginning of training and is particularly useful when fine-tuning pre-trained language models.

For the polarity prediction task, following previous work ^2^, we concatenated a separate embedding generated from the combination of first two utterances (typically the target and its parent), and appended it to the embeddings fed into the classification layer. This helps to incorporate contextual dependency in the decision-making process.

### Evaluation results

Tables 2 and 3 report the classification performance of GPT-4, SoftmaxBERT, and the Multi-Head Attention model across different contextual sampling strategies on the Guest and Kialo datasets. Overall, the results consistently indicate that incorporating structured contextual information improves classification performance over the no-context (0D) setting, with the proposed 2D similarity-based random walk yielding the most robust gains across models and datasets.

#### Overall trends

Across both datasets, moving from 0D to context-aware sampling leads to noticeable improvements in Macro F1 for all three models, highlighting the importance of conversational context for both hate speech detection and polarity prediction. The gains are most evident in improved precision and F1 scores. This indicates that contextual sampling helps models disambiguate cases where the target utterance alone is insufficient to infer intent or polarity.

#### Comparison of sampling strategies

Among the context-aware methods, the 2D-walk consistently outperforms the 1D-walk across all models and both datasets, thanks to the broader contextual coverage. Table 4 reports the fold-wise differences in Macro F1 between the 2D and 1D walks across multiple models and datasets. Notably, the performance difference is non-negative across all folds and models for both datasets, resulting in a positive mean difference in every case. This consistency across folds indicates that the 2D-walk provides a systematic advantage over the 1D strategy, rather than a fold-specific effect. Moreover, the presence of positive mean gains across both GPT-4 and transformer-based classifiers, as well as across both datasets, further demonstrates the robustness of the proposed sampling strategy.

Quantitatively, the transition from 1D to 2D sampling yields consistent gains in Macro F1 across folds and models, ranging from approximately 1.1 to 1.8 percentage points on Guest, and 0.1 to 1.3 percentage points on Kialo (Table 4). While modest in absolute terms, these improvements are systematic and observed across all evaluated models and folds, indicating a stable benefit. Compared to the no-context (0D) setting, moving to 2D sampling yields larger gains, with Macro F1 improving by up to 5.5 percentage points, with the largest increase observed on Kialo.

To further assess the robustness of the observed improvements, we conducted paired *t*-tests across folds ($n = 3$) comparing the Macro F1 scores of the 2D and 1D sampling strategies for each model and dataset. Given the small number of folds, statistical power is limited, and *p*-values should be interpreted with caution, since moderate effects may not reach conventional significance thresholds. In line with the fold-wise results (Table 4), the observed effects (2D > 1D) were consistently in the expected direction, with positive *t* values across all comparisons. For example, GPT-4 on Guest showed a positive but non-significant outcome ($t = 1.77$, $p = 0.109$), while both Multi-Head Attention on Guest ($t = 4.56$, $p = 0.022$) and GPT-4 on Kialo ($t = 4.50$, $p = 0.023$) exhibited significant improvements. Taken together, the positive direction of improvement across independent splits supports the stability of the 2D sampling advantage beyond run-to-run variability.

The 2D-walk also consistently outperforms Random L with up to approximately 6 percentage points in Macro F1, despite using comparable sample size, showing that performance gains cannot be attributed solely to the number of contextual utterances. Random sampling frequently retrieves comments that are topologically distant or semantically irrelevant, diluting useful signals and introducing noise. In contrast, the traversal induced by the 2D-walk ensures that sampled utterances remain both structurally linked to the target and semantically informative.

The Top L baseline which selects the most semantically similar utterances provides a strong point of comparison. While Top L occasionally matches or slightly exceeds the 2D-walk (e.g., GPT-4 on Guest and Kialo), the 2D-walk outperforms it in most cases, particularly for the fine-tuned models. Although the observed differences are generally modest, this pattern suggests that relying on semantic similarity alone is not always sufficient to capture all task-relevant context. By combining semantic guidance with structured traversal and limited stochasticity, the 2D Similarity-based Random Walk is able to incorporate complementary contextual information that may be missed by purely similarity-driven selection. In the subsection “Runtime analysis”, we further show that the Top L algorithm becomes computationally inefficient as graph size increases.

#### Model-specific observations

The consistency of the 2D-walk’s advantage across GPT-4, SoftmaxBERT, and Multi-Head Attention suggests that the benefits of structured contextual sampling are largely model-agnostic. Even for GPT-4, which is capable of strong zero-shot reasoning and long-range dependency modeling, the choice of contextual sampling strategy has a measurable impact on performance. For the lighter-weight transformer models (SoftmaxBERT and Multi-Head Attention), the gains are particularly stable, indicating that well-curated context can partially compensate for architectural simplicity.

An additional observation concerns the behavior of GPT-4 in the no-context (0D) setting. In some cases, GPT-4 (0D) may attain higher recall than context-aware variants, but this is accompanied by a marked drop in precision. Such a precision–recall trade-off is consistent with prior findings on LLMs operating without explicit contextual grounding, where predictions are more strongly influenced by semantic and label priors learned during pre-training, leading to increased sensitivity and over-prediction in ambiguous cases^52^. While this behavior can improve recall, it significantly increases false positives, resulting in lower overall effectiveness. Notably, this pattern is less evident for the simpler discriminative models (SoftmaxBERT and Multi-Head Attention), which exhibit more balanced precision–recall profiles even in the 0D setting.

**Table 2:** Performance comparison of different sampling strategies across different models on the *Guest* dataset. Each reported value represents the mean over 3-fold evaluation. Best sampling strategy per model is bolded. Baselines (Random L, Top L) are italicized. $L=10$.

Model	Macro F1	Precision Non-hate	Precision Hate	Recall Non-hate	Recall Hate	F1 Non-hate	F1 Hate
*GPT-4 (Random L)*	*69.46*	*95.26*	*50.76*	*96.32*	*39.88*	*95.78*	*43.14*
GPT-4 (0D)	72.79	**97.34**	41.09	92.69	**66.87**	94.95	50.62
GPT-4 (1D)	74.34	96.33	51.46	96.05	54.37	96.19	52.50
GPT-4 (2D)	75.43	96.35	55.89	**96.62**	54.37	**96.48**	54.37
*GPT-4 (Top L)*	**76.71**	*96.60*	**56.22**	*96.33*	*58.53*	*96.47*	**56.95**
*SM-BERT (Random L)*	*73.22*	**95.89**	*57.69*	*97.64*	*43.62*	*96.76*	*49.67*
SM-BERT (0D)	72.87	95.24	62.92	98.08	40.25	96.64	49.09
SM-BERT (1D)	74.10	95.45	64.42	98.08	43.03	96.75	51.47
SM-BERT (2D)	**75.92**	95.72	**66.88**	**98.11**	**46.76**	**96.90**	**54.95**
*SM-BERT (Top L)*	*74.39*	*95.51*	*64.27*	*98.05*	*43.69*	*96.76*	*52.01*
*MHA (Random L)*	*73.89*	*95.56*	*65.40*	*97.81*	*43.64*	*96.66*	*51.12*
MHA (0D)	72.66	95.65	53.55	96.42	46.63	96.02	49.30
MHA (1D)	74.38	95.48	65.12	98.02	43.68	96.73	52.04
MHA (2D)	**76.17**	**95.82**	65.89	97.96	**47.93**	96.88	**55.47**
*MHA (Top L)*	*76.03*	*95.74*	**67.05**	**98.10**	*46.89*	**96.91**	*55.14*

*SM-BERT* SoftmaxBERT, *MHA* multi-head attention. Reported values are percentages (%).

**Table 3:** Performance comparison of different sampling strategies across different models on the **Kialo** dataset. Each reported value represents the mean over 3-fold evaluation. Best sampling strategy per model is bolded. Baselines (Random L, Top L) are italicized. $L=3$.

Model	Macro F1	Precision Opposing	Precision Supporting	Recall Opposing	Recall Supporting	**F1** Opposing	F1 Supporting
*GPT-4 (Random L)*	*80.89*	*95.48*	*68.93*	*71.95*	*94.60*	*82.05*	*79.72*
GPT-4 (0D)	77.91	84.89	70.53	**78.88**	77.94	81.77	74.04
GPT-4 (1D)	82.41	95.08	71.28	74.98	93.99	83.81	81.02
GPT-4 (2D)	83.46	**97.34**	71.70	74.91	96.51	**84.65**	82.27
*GPT-4 (Top L)*	**83.49**	*97.20*	**71.80**	*75.00*	**96.61**	*84.65*	**82.33**
*SM-BERT (Random L)*	*81.93*	*83.96*	*80.12*	*85.43*	*78.26*	*84.69*	*79.18*
SM-BERT (0D)	80.47	83.42	77.56	83.02	77.99	83.20	77.74
SM-BERT (1D)	80.94	83.91	77.95	83.26	**78.71**	83.58	78.31
SM-BERT (2D)	**82.24**	**84.26**	**80.41**	**85.61**	78.70	**84.93**	**79.55**
*SM-BERT (Top L)*	*82.17*	*84.18*	*80.37*	*85.59*	*78.57*	*84.88*	*79.46*
*MHA (Random L)*	*82.10*	*84.31*	*80.02*	*85.21*	**78.88**	*84.76*	*79.44*
MHA (0D)	81.38	83.89	78.92	84.27	78.45	84.08	78.69
MHA (1D)	82.11	84.18	80.23	85.46	78.60	84.81	79.41
MHA (2D)	**82.25**	**84.34**	**80.33**	85.50	78.86	**84.92**	**79.59**
*MHA (Top L)*	*82.15*	*84.18*	*80.31*	**85.53**	*78.59*	*84.85*	*79.44*

*SM-BERT* SoftmaxBERT, *MHA* multi-head attention. Reported values are percentages (%)

**Table 4:** Macro F1 differences (in percentage points) between 2D and 1D walks across folds, models, and datasets.

Dataset	Model	Fold 1	Fold 2	Fold 3	Mean Diff. (MD)
Guest	GPT-4	0.00	+1.13	+2.12	**+1.08**
	SM-BERT	+3.98	+0.04	+1.44	**+1.82**
	MHA	+1.07	+1.88	+2.42	**+1.79**
Kialo	GPT-4	+0.78	+1.51	+0.85	**+1.05**
	SM-BERT	+1.25	+1.26	+1.38	**+1.30**
	MHA	+0.20	+0.09	+0.14	**+0.14**

### Runtime analysis

We now analyze the runtime of the five evaluated sampling algorithms to assess their practical utility. In particular, we focus on the degraded efficiency of the Top L sampling baseline as graph size increases, in comparison to our proposed 2D-walk, despite the two approaches achieving similar classification performance.

**Fig. 8:**
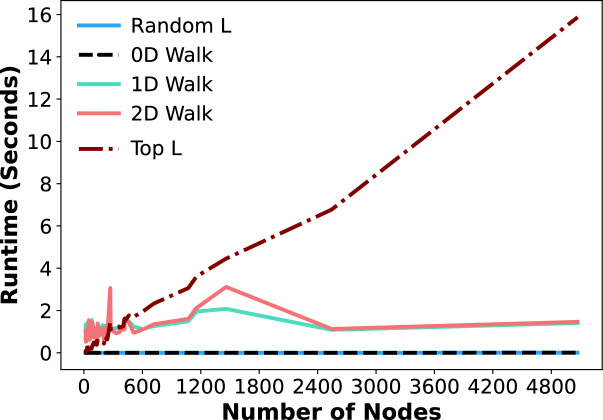
Runtime (in seconds) of five context-sampling algorithms measured on Kialo graphs of increasing size. The Top L baseline exhibits rapidly increasing runtime as graph size grows, indicating poor scalability. In contrast, 1D and 2D Similarity-based Random Walks remain consistently efficient across graph sizes, with minor fluctuations at smaller graphs and stable runtimes for larger ones. 0D walk and Random L incur negligible runtime overhead throughout. Overall, the results highlight the practical efficiency of similarity-based walks compared to exhaustive ranking-based sampling.

We conducted experiments on the Kialo dataset using graphs of varying sizes and recorded the average runtime (in seconds) for each sampling algorithm. As shown in Fig. 8, the runtime of the Top L baseline increases substantially as the number of nodes grows, reaching 16 seconds for graphs with 5,000 nodes. This is because the algorithm computes the semantic similarity between the target utterance and every node in the graph. In practical settings, preparing data for classification models at this rate quickly becomes impractical. In contrast, the runtime of the 2D-walk is considerably more stable. For graphs with fewer than 1,500 nodes, we observe runtime fluctuations between approximately 0 and 3 seconds. This behavior arises because, in relatively small graphs, the walk is more likely to revisit previously visited nodes due to the limited number of alternatives. As the number of nodes increases beyond 1,500, the runtime gradually decreases as the abundance of nodes reduces the likelihood of repetitions or local cycles, eventually stabilizing at around 1 second. This demonstrates that the proposed 2D-walk is more efficient in terms of runtime, hence more practical than the Top L baseline.

We further note that the runtime of the 1D-walk is comparable to that of the 2D-walk, and is slightly lower for graph sizes below 2,500 nodes. As the graph size increases beyond this point, the runtimes of the two algorithms become nearly identical. This observation is consistent with our earlier asymptotic time complexity analysis, which indicates that the 1D and 2D walks share the same time complexity. Finally, we observe that the 0D-walk and Random L baselines exhibit negligible runtime, remaining close to 0 seconds across all graph sizes.

### Discussion on classification performance

These results collectively indicate that effective contextual sampling requires more than selecting many utterances or only the most similar ones. The proposed 2D Similarity-based Random Walk consistently achieves a favorable balance between relevance, diversity, and structural coherence, offering broader contextual coverage than the 1D-walk and resulting in robust performance gains across models and datasets. Importantly, it does so with substantially lower runtime than exhaustive similarity-ranking methods, making it more scalable in practice.

The benefits compared to the 1D-walk are most pronounced on the Guest dataset, where classification decisions are highly sensitive to discourse context and implicit cues. In this setting, broader yet structured exploration enables the model to access complementary information that improves precision and Macro F1. On Kialo, where baseline performance is already strong, the gains are smaller but remain consistent, suggesting that 2D sampling enhances contextual understanding even in near-saturated regimes.

Importantly, the observed improvements are not attributable to increased sample size alone. The 2D-walk outperforms Random L under identical or even smaller context budgets (since it can terminate early), and frequently matches or exceeds the performance of Top L, indicating that optimal context selection depends on the interaction between semantic relevance, conversational structure, and controlled stochastic exploration.

The observed gains for GPT-4, even without task-specific fine-tuning, highlight the practical relevance of 2D sampling for LLMs. Despite GPT-4’s extensive pretraining and generalization capabilities, its performance still benefits substantially–up to nearly 5 percentage points in Macro F1–when supported by better contextual sampling. This suggests that even state-of-the-art LLMs remain sensitive to the structure and relevance of their input context. Incorporating structured, similarity-driven sampling like the 2D-walk can thus serve as a lightweight yet powerful enhancement strategy, especially in downstream applications where context is fragmented or noisy. More broadly, this reinforces the view that optimal performance in real-world language understanding tasks hinges not only on model size but also on the quality of input conditioning^53^, an insight that could inform future prompting and retrieval strategies in LLM pipelines.

## Conclusion

### Research contribution

We introduced the 2D Similarity-based Random Walk, a novel sampling algorithm that enhances the contextual extraction process for social media text classification. By exploring multiple paths within discussion graphs while maintaining the same asymptotic time complexity, the 2D-walk achieves broader coverage across discourse branches than the traditional 1D-walk. Empirically, this expanded traversal guided by semantic relevance to the target utterance, results in samples that are structurally richer and contain more utterances per sample. Furthermore, our extensive evaluations demonstrate that the 2D-walk consistently outperforms 0D, 1D, and random sampling baselines across tasks such as hate speech detection and polarity prediction. The improved classification performance was also validated across multiple transformer-based models, including GPT-4, Multi-Head Attention, and SoftmaxBERT. The reported gains of up to 5% in Macro F1-score, along with improvements in other key metrics, underscore the value of structurally broader sampling for discourse-driven NLP tasks. Overall, this work highlights the importance of intelligent graph traversal strategies for advancing automated understanding of complex online conversations.

### Limitations and future work

While the 2D Similarity-based Random Walk improves contextual sampling and classification performance, it is still guided by semantic similarity alone. Semantic similarity, however, is not always the strongest indicator of relevance, as utterances that are less similar to the target can nevertheless provide crucial contextual cues for interpretation. Moreover, the proposed approach does not incorporate feedback from the downstream model itself, such as its learned representations or attention patterns, when selecting contextual nodes. As a result, the walk may occasionally include utterances that are semantically close to the target but less informative for the specific classification task.

A promising direction for future research is the development of a 2D attention-modulated random walk that incorporates attention signals learned through self-distillation^2^. By using a model’s own learned attention to guide sampling across conversation trees, this approach could focus the contextual window on nodes that are not only structurally or semantically relevant, but also contextually important according to the model itself. This has the potential to produce more informative, fine-tuned samples that further enhance classification performance in complex discourse tasks.

Overall, the success of 2D sampling in both hate speech detection and polarity prediction despite their differing linguistic and structural characteristics underscores its broader utility. Future research could extend this strategy to more complex discourse understanding problems, including dialogue summarization^54^, dialog act classification^55^, or emotion recognition in multi-turn conversations^56^, where effective modeling of context remains a key challenge.

## Supplementary Information

Supplementary Information.

## Data Availability

The data used in this paper can be accessed at the following links: Guest: https://github.com/ellamguest/online-misogyny-eacl2021 Kialo: https://netsys.surrey.ac.uk/datasets/graphnli/

## Code availability

The code used in this paper is publicly available and can be accessed at the following link: https://github.com/socsys/2D-Similarity-based-Random-Walk/ Any questions, requests, or inquiries related to the code should be directed to Zaid Almahmoud at zaid.almahmoud@northwestern.edu.

## References

[1] Eilders, C. & Porten-Cheé, P. Effects of online user comments on public opinion perception, personal opinion, and willingness to speak out: A cross-cultural comparison between germany and south korea. *J. Inf. Technol. Polit.* **20**, 323–337 (2023).

[2] Agarwal, V., Chen, Y. & Sastry, N. Gascom: Graph-based attentive semantic context modeling for online conversation understanding. *Online Soc. Netw. Med.* **43**, 100290 (2024).

[3] Kovács, G., Alonso, P. & Saini, R. Challenges of hate speech detection in social media: Data scarcity, and leveraging external resources. *SN Comput. Sci.* **2**, 95 (2021).

[4] Ramos, G. et al. A comprehensive review on automatic hate speech detection in the age of the transformer. *Soc. Netw. Anal. Min.* **14**, 204 (2024).

[5] Nassif, A. B., Darya, A. M. & Elnagar, A. Empirical evaluation of shallow and deep learning classifiers for Arabic sentiment analysis. *Trans. Asian Low-Resour. Lang. Inf. Process.* **21**, 1–25 (2021).

[6] Raheja, V. & Tetreault, J. Dialogue act classification with context-aware self-attention. arXiv preprint arXiv:1904.02594 (2019).

[7] Jandhyala, V. S. V. Revolutionizing AI interactions: The rise of context-aware systems. *Int. J. Comput. Eng. Technol. (IJCET)* **15**, 776–783 (2024).

[8] Wang, Y., Wang, C., Zhan, J., Ma, W. & Jiang, Y. Text FCG: Fusing contextual information via graph learning for text classification. *Exp. Syst. Appl.* **219**, 119658 (2023).

[9] Zhang, G. et al. Leveraging long context in retrieval augmented language models for medical question answering. *npj Digit. Med.* **8**, 239 (2025). 10.1038/s41746-025-01651-w PMC12048518 40316710

[10] Richardson-Self, L. Woman-hating: On misogyny, sexism, and hate speech. *Hypatia* **33**, 256–272 (2018).

[11] Guest, E. et al. An expert annotated dataset for the detection of online misogyny. In *Proceedings of the 16th Conference of the European Chapter of the Association for Computational Linguistics: Main Volume*. 1336–1350 (2021).

[12] Corazza, M., Menini, S., Cabrio, E., Tonelli, S. & Villata, S. A multilingual evaluation for online hate speech detection. *ACM Trans. Internet Technol. (TOIT)* **20**, 1–22 (2020).

[13] Frenda, S. et al. Sarcasm and Implicitness in Abusive Language Detection: A Multilingual Perspective (2022).

[14] Kialo: A Platform for Thoughtful Debate (2025). https://www.kialo.com. Accessed 06 Apr 2025.

[15] Agarwal, V., Joglekar, S., Young, A. P. & Sastry, N. Graphnli: A graph-based natural language inference model for polarity prediction in online debates. In *The ACM Web Conference (TheWebConf)* (2022).

[16] Pérez, J. M. et al. Assessing the impact of contextual information in hate speech detection. *IEEE Access* **11**, 30575–30590 (2023).

[17] Yu, X., Blanco, E. & Hong, L. Hate speech and counter speech detection: Conversational context does matter. arXiv preprint arXiv:2206.06423 (2022).

[18] OpenAI. GPT-4 Language Model (API). https://openai.com/index/gpt-4 (2023). Accessed 18 May 2025.

[19] Hugging Face. all-MiniLM-L6-v2 (Sentence Transformer Model). https://huggingface.co/sentence-transformers/all-MiniLM-L6-v2 (2021). Accessed 18 May 2025.

[20] Zhang, Z., Li, J., Zhu, P., Zhao, H. & Liu, G. Modeling multi-turn conversation with deep utterance aggregation. arXiv preprint arXiv:1806.09102 (2018).

[21] Chen, Y., Wu, L. & Zaki, M. J. Graphflow: Exploiting conversation flow with graph neural networks for conversational machine comprehension. arXiv preprint arXiv:1908.00059 (2019).

[22] Hu, J., Wu, L., Chen, Y., Hu, P. & Zaki, M. J. Graphflow+: Exploiting conversation flow in conversational machine comprehension with graph neural networks. *Mach. Intell. Res.* **21**, 272–282 (2024). https://link.springer.com/article/10.1007/s11633-023-1421-0.

[23] Ghosal, D., Majumder, N., Poria, S., Chhaya, N. & Gelbukh, A. Dialoguegcn: A graph convolutional neural network for emotion recognition in conversation. arXiv preprint arXiv:1908.11540 (2019).

[24] Wang, B. et al. Hierarchically stacked graph convolution for emotion recognition in conversation. *Exp. Syst. Appl.* **206**, 117698 (2023). https://www.sciencedirect.com/science/article/pii/S0950705123000357.

[25] Li, J., Wang, X., Lv, G. & Zeng, Z. Graphcfc: A directed graph based cross-modal feature complementation approach for multimodal conversational emotion recognition. arXiv preprint arXiv:2207.12261 (2022).

[26] Alfarizy, G. & Mandala, R. Verification of unanswerable questions in the question answering system using sentence-bert and cosine similarity. In *2022 9th International Conference on Advanced Informatics: Concepts, Theory and Applications (ICAICTA)*. 1–6 (IEEE, 2022).

[27] Santander-Cruz, Y., Salazar-Colores, S., Paredes-García, W. J., Guendulain-Arenas, H. & Tovar-Arriaga, S. Semantic feature extraction using sbert for dementia detection. *Brain Sci.* **12**, 270 (2022). 35204032 10.3390/brainsci12020270 PMC8870383

[28] Bithel, S. & Malagi, S. S. Unsupervised identification of relevant prior cases. arXiv preprint arXiv:2107.08973 (2021).

[29] Gleich, D. F. Pagerank beyond the web. *SIAM Rev.* **57**, 321–363 (2015).

[30] Riascos, A. P., Boyer, D., Herringer, P. & Mateos, J. L. Random walks on networks with stochastic resetting. *Phys. Rev. E* **101**, 062147 (2020). 32688619 10.1103/PhysRevE.101.062147

[31] Izyumov, A. & Samokhin, K. Field theory of self-avoiding walks in random media. *J. Phys. A Math. Gen.* **32**, 7843 (1999).

[32] Madras, N. & Slade, G. *The Self-Avoiding Walk* (Springer, 2013).

[33] Davidson, T., Warmsley, D., Macy, M. & Weber, I. Automated hate speech detection and the problem of offensive language. *Proc. Int. AAAI Conf. Web Soc. Med.* **11**, 512–515 (2017).

[34] Warner, W. & Hirschberg, J. Detecting hate speech on the world wide web. In *Proceedings of the Second Workshop on Language in Social Media*. 19–26 (2012).

[35] Malik, J. S., Qiao, H., Pang, G. & van den Hengel, A. Deep learning for hate speech detection: A comparative study. *Int. J. Data Sci. Anal.* 1–16 (2024).

[36] Cao, R., Lee, R. K.-W. & Hoang, T.-A. Deephate: Hate speech detection via multi-faceted text representations. In *Proceedings of the 12th ACM Conference on Web Science*. 11–20 (2020).

[37] Mozafari, M., Farahbakhsh, R. & Crespi, N. A bert-based transfer learning approach for hate speech detection in online social media. In *International Conference on Complex Networks and Their Applications*. 928–940 (Springer, 2019).

[38] Liu, D., Wang, M. & Catlin, A. G. Detecting anti-semitic hate speech using transformer-based large language models. arXiv preprint arXiv:2405.03794 (2024).

[39] Mandal, A., Roy, G., Barman, A., Dutta, I. & Naskar, S. K. Attentive fusion: A transformer-based approach to multimodal hate speech detection. arXiv preprint arXiv:2401.10653 (2024).

[40] Esuli, A. & Sebastiani, F. Sentiwordnet: A publicly available lexical resource for opinion mining. In *Proceedings of LREC*. 417–422 (2006).

[41] Miao, Y., Su, J., Liu, S. & Wu, K. So-cal based method for Chinese sentiment analysis. In *Informatics and Management Science IV*. 345–351 (Springer, 2013).

[42] Barnes, J., Øvrelid, L. & Velldal, E. Sentiment analysis is not solved! Assessing and probing sentiment classification. arXiv preprint arXiv:1906.05887 (2019).

[43] Garg, K. & Caragea, C. Stanceformer: Target-aware transformer for stance detection. arXiv preprint arXiv:2410.07083 (2024).

[44] Hassan, A. & Radev, D. Identifying text polarity using random walks. In *Proceedings of the 48th Annual Meeting of the Association for Computational Linguistics*. 395–403 (2010).

[45] Montejo-Ráez, A., Martínez-Cámara, E., Martin-Valdivia, M. T. & López, L. A. U. Random walk weighting over sentiwordnet for sentiment polarity detection on twitter. In *Proceedings of the 3rd Workshop in Computational Approaches to Subjectivity and Sentiment Analysis*. 3–10 (2012).

[46] Perozzi, B., Al-Rfou, R. & Skiena, S. Deepwalk: Online learning of social representations. In *Proceedings of the 20th ACM SIGKDD International Conference on Knowledge Discovery and Data Mining*. 701–710 (2014).

[47] Reimers, N. & Gurevych, I. Sentence-bert: Sentence embeddings using siamese bert-networks. arXiv preprint arXiv:1908.10084 (2019).

[48] Björklund, A., Husfeldt, T. & Khanna, S. Approximating longest directed paths and cycles. In *Automata, Languages and Programming: 31st International Colloquium, ICALP 2004, Turku, Finland, July 12-16, 2004. Proceedings 31*. 222–233 (Springer, 2004).

[49] Pardey, J. & Rautenbach, D. Vertex degrees close to the average degree. *Discrete Math.* **346**, 113599 (2023).

[50] Vaswani, A. et al. Attention is all you need. In *Advances in Neural Information Processing Systems*. Vol. 30 (2017).

[51] Tarjan, R. Depth-first search and linear graph algorithms. *SIAM J. Comput.* **1**, 146–160 (1972).

[52] Zhao, Z., Wallace, E., Feng, S., Klein, D. & Singh, S. Calibrate before use: Improving few-shot performance of language models. In *International Conference on Machine Learning*. 12697–12706 (PMLR, 2021).

[53] Park, D., An, G.-T., Kamyod, C. & Kim, C. G. A study on performance improvement of prompt engineering for generative AI with a large language model. *J. Web Eng.* **22**, 1187–1206 (2023).

[54] Jia, Q., Liu, Y., Ren, S. & Zhu, K. Q. Taxonomy of abstractive dialogue summarization: Scenarios, approaches, and future directions. *ACM Comput. Surv.* **56**, 1–38 (2023).

[55] Witzig, P., Constantin, R., Kovacevic, N. & Wampfler, R. Multimodal dialog act classification for digital character conversations. In *Proceedings of the 6th ACM Conference on Conversational User Interfaces*. 1–14 (2024).

[56] Ma, H., Wang, Z., Zhou, X., Zhou, G. & Zhou, Q. Emotion recognition with conversational generation transfer. *Trans. Asian Low-Resour. Lang. Inf. Process.* **21**, 1–17 (2022).

