# Supplementary material for: Modeling discourse structure with 2D similarity-based random walks for improved understanding of online conversations
Source: Sci Rep. 2026 Mar 12;16:17216. doi: 10.1038/s41598-026-43577-7 (PMC13234104; doi:10.1038/s41598-026-43577-7)
Supplement: Supplementary file 1 — Supplementary Information. [file 41598_2026_43577_MOESM1_ESM.pdf]

Modeling discourse structure with 2D Similarity-based Random Walks for improved understanding of online conversations

Zaid Almahmoud, Vibhor Agarwal, Rana Mahmoud, and Nishanth Sastry

Supplementary Methods

Controlling for sample size

We examined whether the observed improvements from 2D-walks over 1D-walks are solely due to the increased sample size. To this end, we repeated the evaluation experiments after equalizing the number of utterances per sample. Specifically, for each pair of 1D and 2D walks, we trimmed the sequences to the minimum length among the two walks to ensure that both walks contributed the same number of utterances.

The classification results of GPT-4, averaged over three experimental runs, show that the 2D-walk consistently outperforms the 1D-walk even after equalizing the sample length. On the Guest dataset, the 2D-walk achieves a Macro F1 score of 68.13%, compared to 61.83% for the 1D-walk, corresponding to an improvement of over six percentage points. On the Kialo dataset, the 2D-walk also yields a higher Macro F1 score (79.59%) than the 1D-walk (79.09%), indicating a smaller but consistent gain. The results confirm that the performance gains of 2D-walks persist even when sample size is controlled. While increasing the number of utterances can contribute to the improved performance, since it increases the likelihood of collecting relevant context, the results indicate that the structural advantages of the 2D-walk and its ability to capture broader contextual information also play a key role. This suggests that the improvements are not merely a function of sample size and reflects the richer contextual information encoded by the 2D sampling strategy.

Abstention-enabled GPT-4 predictions

To assess whether forced binary outputs encourage overconfident predictions, we repeated the evaluation of GPT-4 (0D) model while allowing the model to abstain when not sure by outputting an explicit -1 label. On the Kialo dataset, the model abstained in 13 out of 400 instances (3.25%). Importantly, 6 of these abstentions (46.2%) coincided with misclassifications under forced binary (0/1) outputs, indicating that nearly half of the abstentions correctly flagged cases where binary predictions were incorrect.

All flagged instances corresponded to false negatives. That is, these were cases in which the ground-truth label was positive (the utterance supports the utterance it is replying to), but the forced binary prediction incorrectly assigned a negative (opposing) label. This asymmetric pattern suggests that abstention primarily occurs when the model encounters statements that are formally phrased, non-inflammatory, and require implicit inferential reasoning rather than surface-level lexical cues. When constrained to produce a binary decision, the model tends to default to the negative class, whereas the option to abstain allows it to signal uncertainty instead of committing to an incorrect label.

A qualitative inspection of the abstained instances (Supplementary Table S1) confirms this behavior. For example, several flagged cases involve normative or philosophical claims (*e.g.,* arguments about self-reliance, institutional responsibility, or historical commemoration) that lack explicit markers associated with the positive class. In these cases, the correct label depends on broader argumentative context, which was not provided to the model. Interestingly, when we provided the 2D-walk context to the model, all the 6 misclassified instances were correctly classified.

Supplementary Table S1: Flagged abstention cases on the Kialo dataset where GPT-4 abstained when allowed (-1) and the forced GPT-4 binary prediction resulted in a misclassification. All cases correspond to false negatives (ground truth = 1 (*supporting*), prediction = 0 (*attacking*)).

Utterance	Replying to	Label	Prediction
People should not rely on others for their own self-happiness and instead work on trying to create that for themselves.	Just because something has a good effect, does not make it “right”.	1 (supporting)	0 (attacking)
Religious organizations have to debate their claims, because of their universal vocation. To be able to stand in a plural contest and argue has its specific pedagogical value.	Religious organizations build schools (and universities), orphanages, and other services for community benefits.	1 (supporting)	0 (attacking)
In countries where birth control and abortions are illegal, due to the biological attachment women have to children, it is more likely for a woman to deal with the trauma and burden of an unlicensed child than a man.	Such a test would adversely affect some groups in society more than others.	1 (supporting)	0 (attacking)
A coalition government can find a single person who would not be divisive to fill the role of the unitary president.	The reason why new presidents constantly undo the previous president’s actions is because the current FPTP voting method maintains a polarizing two-party system.	1 (supporting)	0 (attacking)
Is it still okay to venerate George Washington and Thomas Jeffer-<br>son?	People are talking about removing George Washington statues and public references.	1 (supporting)	0 (attacking)
The prominent cause of violence in the US is that people have become	Access to firearms is not the true cause of violence.	1 (supporting)	0 (attacking)

desensitised to loss of life.			

These findings reveal that binary classification encourages overconfident predictions in cases where the available information is insufficient. This behavior is consistent with prior observations that pretrained language models are optimized to maximize accuracy under evaluation settings that reward guessing, as this leads to a better performance [57]. Allowing an explicit "I don't know" option does not eliminate errors, but it effectively highlights a substantial subset of cases where binary predictions are most likely to be incorrect. However, abstention should be interpreted as a diagnostic signal rather than a substitute for binary decision-making: in our experiments, providing additional contextual information corrected these misclassifications in the observed cases. This suggests that uncertainty in such instances is largely attributable to missing context, and that enriching the input can be more effective than forcing a binary decision when the model lacks sufficient evidence.

Importantly, we found that providing 2D-walk context reduced the number of abstentions from 13 to just 2 instances. This substantial drop indicates that uncertainty in GPT-4's predictions is largely driven by insufficient or impoverished context rather than inherent model limitations. Supplying structurally richer and semantically aligned contextual evidence through 2D-walk enables the model to resolve ambiguities that would otherwise trigger abstention or erroneous binary decisions. These results highlight the central role of context quality in mitigating uncertainty, improving decision confidence, and reducing errors, reinforcing the view that principled contextual sampling is a key lever for enhancing LLM reliability in challenging classification settings.

Context order analysis

To structure the contextual input provided to GPT-4, we ordered the set of utterances collected by the sampling methods using a DFS traversal over the sampled walk. Starting from each root in the walk, DFS recursively explores semantically connected utterances along a single branch before backtracking, producing a coherent sequence that reflects local conversational structure. This step does not alter the content of the sampled context but only its presentation order, resolving the lack of ordering introduced by the sampling process. Since LLMs are sensitive to input sequencing, imposing a deterministic and semantically grounded ordering helps the model better integrate contextual information and reason over related utterances [58].

To assess the effect of contextual ordering, we compare DFS-ordered context with a random ordering that follows the sequence in which utterances are sampled. Supplementary Table S2 compares GPT-4 classification performance on Guest dataset when contextual utterances from the 2D-walk are structured using a DFS ordering versus a random ordering corresponding to the original order of the walk. As shown in the table, using a random order degrades GPT-4 performance, most notably resulting in a reduction of approximately 4 points in Macro F1. This decline is accompanied by significantly lower precision, recall and F1 scores for the hate speech class (class 1), suggesting that disrupting semantic coherence affects minority-class detection.

We note that when multiple utterances occur at the same timestamp (or at the same tree level), DFS provides a consistent and deterministic mechanism for ordering them by fully exploring the children of one branch before moving to the next. This preserves sequential coherence of the conversations as much as possible based on discourse connectivity. In contrast, random ordering introduces additional variability in the contextual sequence. These results indicate that GPT-4 is sensitive not only to the content of contextual utterances but also to their relative ordering.

Supplementary Table S2: GPT-4 classification performance on the Guest (hate speech) dataset when contextual utterances sampled by the 2D-walk are ordered using DFS vs. a random order.

Ordering Strategy	Macro F1	Prec. 0	Prec. 1	Recall 0	Recall 1	F1 0	F1 1
**DFS-ordered context**	**75.43**	**96.35**	**55.89**	96.62	**54.37**	**96.48**	**54.37**
**Random (walk order)**	71.87	95.82	50.00	**96.63**	45.44	96.22	47.53

Reported values are percentages (%) that correspond to mean performance across folds.

Sample size analysis

We next investigate whether increasing the number of utterances included per sample consistently improves classification performance, to address scenarios where accurate prediction depends on local or short-range contextual information. To this end, we conduct an experiment on the Guest and Kialo datasets, varying the random walk length L and reporting the Macro F1 score of GPT-4 on the downstream classification task for both 1D and 2D walks. The results are shown in Supplementary Fig. S1.

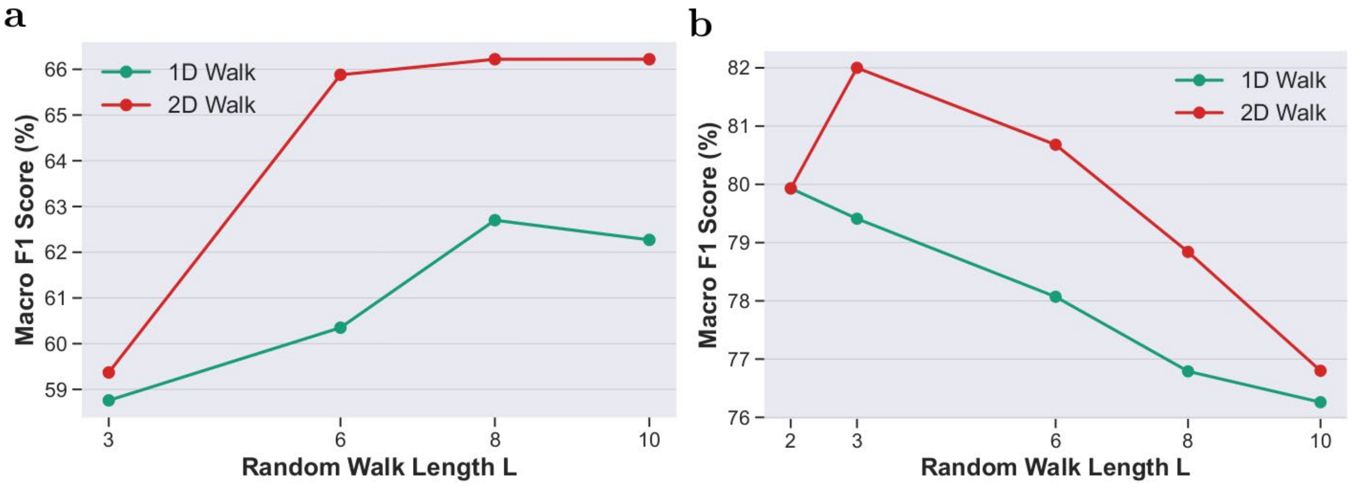

Supplementary Figure S1: Macro F1 score as a function of random walk length L for 1D and 2D sampling strategies on two datasets. **a** Results on the Guest dataset show a strong performance gain as L increases, with the 2D-walk achieving consistently higher Macro F1 scores and saturating at longer walk lengths. **b** Results on the Kialo dataset indicate peak performance at shorter walk lengths, followed by a gradual decline as L increases, suggesting that stance-relevant information is more locally concentrated. Across both datasets, the 2D-walk outperforms the 1D-walk for all values of L, highlighting the robustness of multi-dimensional context sampling.

For the Guest dataset (Supplementary Fig. S1**a**), performance improves noticeably as L increases from 3 to 6 for both walk strategies, with a substantially steeper gain for the 2D-walk. The Macro F1 score for the 2D-walk rises sharply at $L = 6$ and then saturates at $L = 8$ and $L = 10$, indicating diminishing returns from adding longer contextual sequences beyond this point. In contrast, the 1D-walk exhibits a more gradual improvement and shows a slight decline at $L = 10$, suggesting that excessive linear context may introduce noise rather than additional discriminative information. Across all values of L, the 2D-walk consistently outperforms the 1D-walk, highlighting the benefit of incorporating multi-dimensional neighborhood structure when sampling utterances.

In the case of the Kialo dataset (Supplementary Fig. S1**b**), a different trend emerges. Both 1D and 2D walks achieve their highest Macro F1 scores at shorter walk lengths, with peak performance observed at $L = 3$ for the 2D-walk and at $L = 2$ for the 1D-walk. As L increases beyond these points, performance

steadily declines for both strategies, indicating that longer walks may dilute the stance-relevant signal with less pertinent contextual information. Nevertheless, the 2D-walk maintains a consistent performance advantage over the 1D-walk across all walk lengths, although the gap between the two narrows as L increases. In our main experiments (Tables 2 and 3), we selected a fixed walk length L that yields peak performance for the 2D-walk ($L = 10$ for Guest and $L = 3$ for Kialo) to ensure a fair comparison under a fixed computational budget.

Interestingly, the Guest and Kialo datasets exhibit different sensitivity to walk length. While increasing walk length monotonically improves performance on Guest, Kialo shows a performance peak followed by a gradual decline. This difference reflects an interaction between walk length and the nature of the utterances. The depth and breadth of a dataset define the potential amount of context that longer walks can access; however, in practice, the communicative style of the posts and comments mediates this effect. Guest utterances are short, informal, and context-dependent, meaning that longer walks provide valuable additional information, resulting in monotonic performance gains (*e.g.,* "Exactly. That's the problem." requires prior context). In contrast, Kialo utterances are formal, longer, and largely self-contained. Even though Kialo graphs have greater depth and breadth (Table 1) — which could theoretically make longer walks more informative — additional walk length often introduces redundancy or noise, leading to the observed performance peak (*e.g.,* "Universal basic income may reduce poverty; however, it risks disincentivizing workforce participation" is already interpretable without further context). These observations suggest that optimal walk length depends not only on graph topology but also on discourse characteristics of the dataset.

Overall, these results suggest that the optimal walk length is dataset-dependent and closely tied to the nature of the discourse structure. While longer walks benefit the Guest dataset up to a saturation point by providing richer context, reasonably shorter walks are more effective for Kialo, where stance information appears to be more locally concentrated. Importantly, the consistent superiority of 2D-walks across both datasets and all values of L highlights the robustness of multi-dimensional sampling in capturing informative contextual dependencies.

While the above analysis highlights the importance of selecting an appropriate walk length L, it also reveals a practical limitation of fixed-length sampling strategies: the optimal value of L is highly dataset-dependent. As shown in Supplementary Fig. S1, longer walks are beneficial for Guest up to a saturation point, whereas shorter walks are clearly preferable for Kialo. Consequently, determining an optimal L requires running separate ablation studies for each dataset, which increases experimental cost and reduces the adaptability of the method when applied to new domains or unseen discourse structures.

To address this limitation, we propose a probabilistic termination mechanism that dynamically controls the walk length based on semantic relevance. At each transition step t, we compute a semantic similarity score $s_t \in [0,1]$ between the current utterance and the target utterance. The walk terminates with probability

$$p_{term}(t) = 1 - s_t$$

such that transitions to semantically less relevant utterances are increasingly likely to end the walk. Equivalently, the continuation probability is proportional to semantic relevance, encouraging longer walks only when contextual coherence is preserved. This adaptive termination strategy removes the need to predefine a fixed walk length, allowing the model to automatically balance contextual richness and noise suppression. As a result, the sampling process becomes both more computationally efficient and more robust across datasets with differing discourse characteristics, while preserving the performance gains associated with multi-dimensional (2D) walks.

Retry budget analysis

To assess the robustness of the retry budget parameter M, we ran an experiment on the Guest dataset, analyzing its effect relative to the walk length L by varying $M \in \{L/2, L, 1.5L, 2L\}$ while keeping all other settings fixed. Supplementary Figure S2 summarizes the impact of M on structural properties of the sampled context, semantic relevance, computational cost, and downstream classification performance for both 1D and 2D Similarity-based Random Walks.

As shown in Supplementary Figs. S2**a** and S2**b**, increasing M leads to an increase in both walk depth and breadth, as additional retries allow the walk to escape local dead-ends and explore alternative branches. However, this increase slows down once $M >= L$, indicating that further increases in M provide diminishing gains in depth and breadth.

Supplementary Figure S2**c** reports the average semantic similarity between sampled utterances and the target utterance v_0. For both walks, semantic similarity generally decreases as M increases, reflecting the inclusion of more distant and less directly related context. An exception is observed for the 1D-walk, where similarity initially increases up to $M = L$. This behavior arises because small retry budgets cause the 1D-walk to terminate prematurely due to its higher susceptibility to local cycles, limiting its ability to collect meaningful context. Allowing a larger M initially mitigates this effect, before similarity begins to decrease as the walk expands further.

The effect of M on sample size is shown in Supplementary Fig. S2**d**. As expected, increasing M allows the walk to collect more utterances, with sample size growing steadily up to $M = L$, then slowing down and approaching saturation.

Runtime behavior is illustrated in Supplementary Fig. S2**e**. Runtime generally increases with M for both walk variants, with a more pronounced effect for the 1D-walk. This is because larger retry budgets allow the 1D-walk to remain active longer in locally cyclic regions, repeatedly attempting transitions before termination.

Most importantly, Supplementary Fig. S2**f** shows the impact of M on downstream Macro F1 performance. For the 2D-walk, Macro F1 increases with M and peaks at $M = L$, after which performance declines. For the 1D-walk, the peak occurs later at $M = 1.5L$, followed by a sharp decrease. This trend reflects the trade-off between semantic relevance and contextual coverage: while larger M increases sample size, excessive retries introduce context that is less semantically aligned with the target utterance, which ultimately harms classification performance.

Overall, these results indicate that neither semantic similarity nor sample size alone is sufficient for optimal performance. Instead, effective classification requires a balance between collecting enough contextual information and maintaining semantic relevance to the target utterance. Based on this analysis, we set $M = L$ in all experiments to provide a fixed and fair budget across both walk variants. Importantly, even when the 1D-walk is allowed a larger retry budget, it does not reach the Macro F1 performance achieved by the 2D-walk at $M = L$ (as shown in Supplementary Fig. S2**f**), further highlighting the advantage of the proposed 2D traversal strategy.

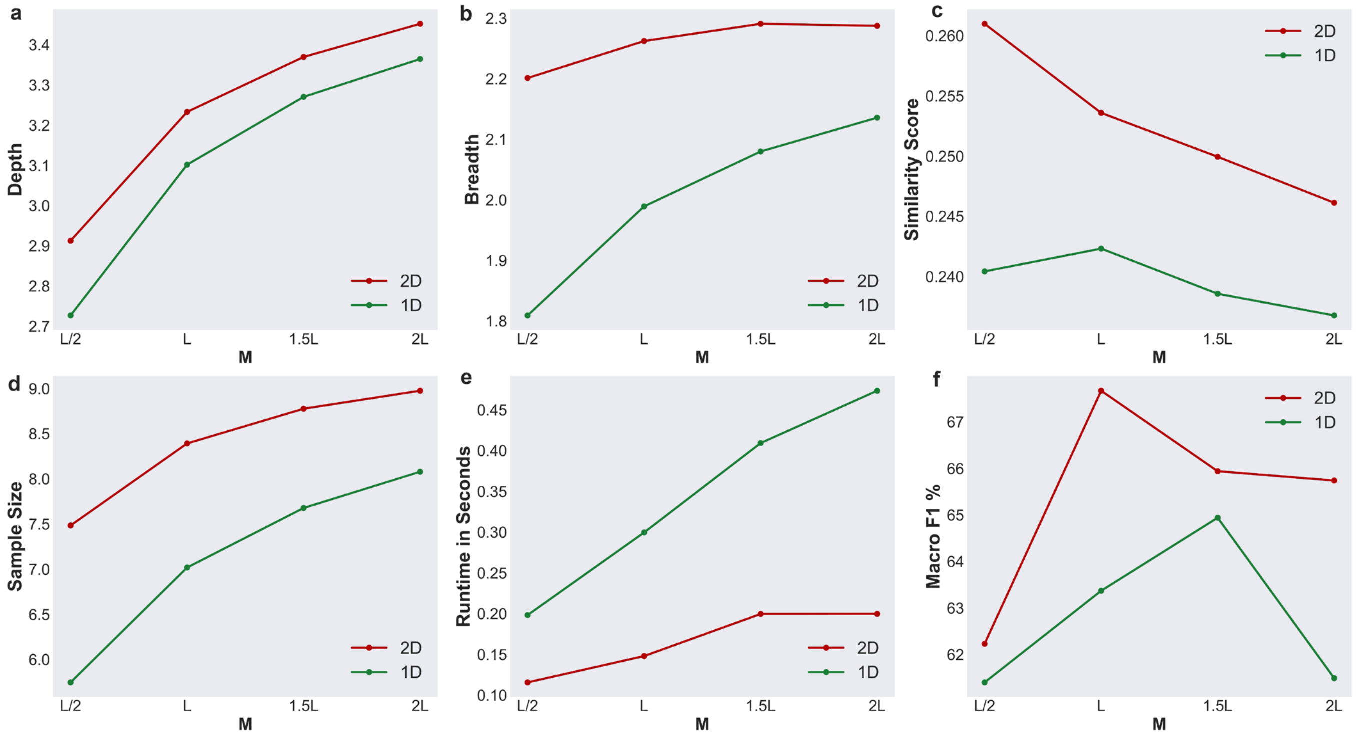

Supplementary Figure S2: Retry budget (M) analysis for 1D and 2D Similarity-based Random Walks on the Guest dataset. The figure analyzes the effect of varying the retry budget M relative to the walk length $L = 10$ on structural, semantic, computational, and performance-related properties of the sampled context. Panels **a** and **b** show average walk depth and breadth, which increase with larger M and with a slower increase or saturation beyond $M = L$. Panel **c** reports the average semantic similarity between sampled utterances and the target utterance, which generally decreases as M increases, except for an initial rise in the 1D-walk due to reduced premature termination. Panel **d** shows that sample size increases with M before saturating, while panel **e** illustrates the increase in runtime, particularly for the 1D-walk, as larger retry budgets prolong exploration in locally cyclic regions. Panel **f** presents downstream Macro F1 performance, which peaks at $M = L$ for the 2D-walk and at $M = 1.5L$ for the 1D-walk before declining, reflecting the trade-off between semantic relevance and contextual coverage. Overall, the results support $M = L$ as an effective and robust setting that balances performance and efficiency.

References

[57] Kalai, A. T., Nachum, O., Vempala, S. S. & Zhang, E. Why language models hallucinate. *arXiv preprint arXiv:2509.04664* (2025).

[58] Li, W., Wang, Y., Wang, Z. & Shang, J. Order matters: Rethinking prompt construction in in-context learning. *arXiv preprint arXiv:2511.09700* (2025).
